# Vortex dynamics in the two-dimensional BCS-BEC crossover

**DOI:** 10.1038/s41467-022-34756-x

**Published:** 2022-11-16

**Authors:** Max Heyl, Kyosuke Adachi, Yuki M. Itahashi, Yuji Nakagawa, Yuichi Kasahara, Emil J. W. List-Kratochvil, Yusuke Kato, Yoshihiro Iwasa

**Affiliations:** 1 Quantum-Phase Electronics Center and Department of Applied Physics, University of Tokyo, Tokyo, Japan; 2 Department of Chemistry, Department of Physics & IRIS Adlershof, Humboldt-Universität zu Berlin, Zum Großen Windkanal 2, Berlin, Germany; 3 Nonequilibrium Physics of Living Matter RIKEN Hakubi Research Team, RIKEN Center for Biosystems Dynamics Research, Kobe, Japan; 4 RIKEN Interdisciplinary Theoretical and Mathematical Sciences Program, Wako, Japan; 5 Department of Physics, Kyoto University, Kitashirakawa Oiwakecho, Kyoto, Japan; 6 Helmholtz-Zentrum für Materialien und Energie GmbH, Hahn-Meitner-Platz 1, Berlin, Germany; 7 Department of Basic Science, University of Tokyo, Tokyo, Japan; 8 RIKEN, Center for Emergent Matter Science, Wako, Japan

**Keywords:** Superconducting properties and materials, Bose-Einstein condensates

## Abstract

The Bardeen–Cooper–Schrieffer (BCS) condensation and Bose–Einstein condensation (BEC) are the two limiting ground states of paired Fermion systems, and the crossover between these two limits has been a source of excitement for both fields of high temperature superconductivity and cold atom superfluidity. For superconductors, ultra-low doping systems like graphene and Li_*x*_ZrNCl successfully approached the crossover starting from the BCS-side. These superconductors offer new opportunities to clarify the nature of charged-particles transport towards the BEC regime. Here we report the study of vortex dynamics within the crossover using their Hall effect as a probe in Li_*x*_ZrNCl. We observed a systematic enhancement of the Hall angle towards the BCS-BEC crossover, which was qualitatively reproduced by the phenomenological time-dependent Ginzburg-Landau (TDGL) theory. Li_*x*_ZrNCl exhibits a band structure free from various electronic instabilities, allowing us to achieve a comprehensive understanding of the vortex Hall effect and thereby propose a global picture of vortex dynamics within the crossover. These results demonstrate that gate-controlled superconductors are ideal platforms towards investigations of unexplored properties in BEC superconductors.

## Abstract (web summary)

The crossover between the limiting ground states of fermionic systems, the so called BCS-BEC crossover, is of interest to superconductor and ultracold atomic gases communities. The authors study the dynamics of superconducting vortices in the crossover regime via the Hall response in a 2D superconductor Li_x_ZrNCl.

## Introduction

**Fig. 1:**
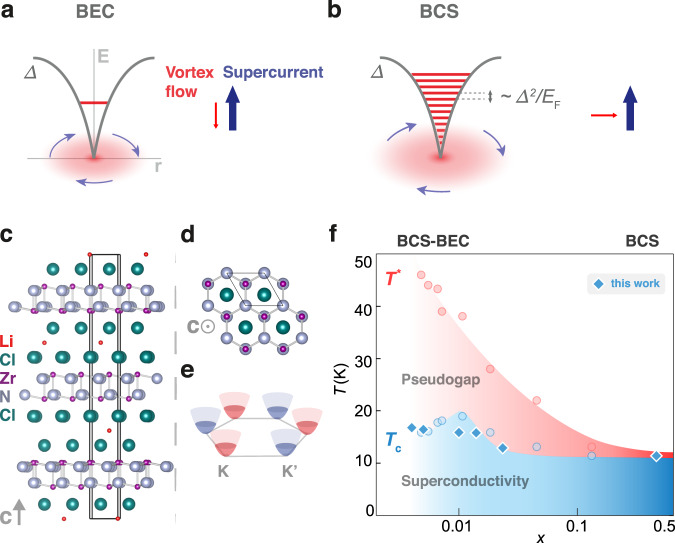
Properties of superconducting Li_*x*_ZrNCl. **a**, **b** Caroli-de Gennes-Matricone (CdGM) schematic of the quantized state(s) in the vortex core in the BEC **a** and BCS limit **b** are depicted. The vortex core is shaded in red, and the axis labels E and r correspond to energy and distance, respectively. The directions of supercurrent and vortex flow are indicated. In the BEC limit, the vortex motion is dissipationless and the vortex flow is nearly anti-parallel to the supercurrent, while in the BCS limit the vortex motion is nearly perpendicular to the supercurrent. Side **c** and top **d** view of the Li_*x*_ZrNCl crystal structure. The solid black lines represent the hexagonal unit cell. **e** Schematic of the simple parabolic conduction bands of Li_*x*_ZrNCl, located at the corners of the hexagonal Brillouin zone (K and K’ points). **f** The BCS-BEC crossover phase diagram of Li_*x*_ZrNCl^9^ with data points from this work superimposed. *T*_c_ was determined by the temperature at which the resistance is half the normal state value at 30 K, which is close enough to the Berezinskii–Kosterlitz–Thouless transition for 2D superconductors^9^. The gap-opening temperature T^*^ was determined in the previous study^9^, and the regime between *T*_c_ and T^*^ forms the pseudo-gap state.

The crossover between the two limiting ground states of Fermion systems—the BCS and BEC state—has attracted continuous interest both theoretically and experimentally from the communities of ultracold atomic gases and superconductors^1–3^. The first experimental realization of the BCS-BEC crossover was achieved in ultracold atomic gases^4^ starting from the BEC side, while the approach with superconductors from the BCS side^5^ has become active since recent discoveries of suitable materials including FeSe^6^, twisted graphene^7,8^, and Li_*x*_ZrNCl^9^. The two BCS-BEC crossover systems, twisted trilayer graphene and Li_*x*_ZrNCl, are highly two-dimensional (2D), and the carrier density can be controlled by a gate voltage. The tunable carrier density is highly advantageous to observe how the system evolves from the BCS- to the BEC-limit. In fact, both systems approached the crossover regime by reducing the carrier density and reached *T*_c_/*T*_F_~1/8, where *T*_c_ and *T*_F_ are the critical temperature and the Fermi temperature, respectively. Since *T*_c_/*T*_F_ = 1/8 is the upper limit for 2D systems^10,11^ in the crossover, it confirms the successful approach of the 2D BCS-BEC crossover. By approaching the crossover towards the BEC-limit, the coupling strength described as the ratio of superconducting gap *Δ* to Fermi energy *E*_F_ increased^9^. One of the intriguing phenomena related to the enhancement of *Δ*/*E*_F_ is the dynamics of superconducting vortices. Figure 1a, b shows a comparison of the energy levels inside the vortices for the BEC and BCS limit. Inside the vortex core, the quasiparticle states are confined and quantized. According to the Caroli-de Gennes-Matricon picture^12^, the energy level spacing is in the order of *Δ*^2^/*E*_F_. In the BCS limit, *Δ*^2^/*E*_F_ is so small that the energy spectrum is almost continuous (Fig. 1b), and thus the quasiparticles in the core are easily scattered, resulting in energy dissipative vortex motion. In the BEC limit, on the other hand, the energy level spacing is large enough to form a single quantized level^13^ (Fig. 1a). This large level spacing renders the vortex motion dissipationless since the quasiparticles in the core cannot be scattered. The above difference results in contrasting vortex motions: the vortex in the BCS regime moves perpendicularly to the net supercurrent (Fig. 1b, arrows), while the vortex in the BEC regime moves parallel to the superfluid velocity, i.e., anti-parallel to the net supercurrent (Fig. 1a, arrows). In the BEC regime, the motion of vortices antiparallel (parallel) to the background current density **J** (superfluid velocity) can be understood as follows: The vortex motion in the presence of the magnetic field **B** induces the macroscopic electric field ${{{{{\bf{E}}}}}}={{{{{\bf{B}}}}}}\times {{{{{{\bf{v}}}}}}}_{{{\bf{v}}}}$, where ${{{{{{\bf{v}}}}}}}_{{{\bf{v}}}}$ is the vortex velocity. In the absence of dissipation in the steady state, ${{{{{\bf{E}}}}}}={{{{{\bf{B}}}}}}\times {{{{{{\bf{v}}}}}}}_{{{\bf{v}}}}$should be perpendicular to ${{{{{\bf{J}}}}}}$ to avoid acceleration of the supercurrent. Thus ${{{{{\bf{J}}}}}}\parallel {{{{{{\bf{v}}}}}}}_{{{\bf{v}}}}$ follows. Hence, the dissipationless nature of the vortex core in the BEC regime (Fig. 1a) brings about vortex flow parallel or antiparallel to the supercurrent flow. This vortex flow parallel to the supercurrent has never been observed experimentally, but in the case of superfluidity of charge neutral bosons of ^4^He, the parallel vortex flow was nicely demonstrated in the movie of ref. 14 (for more details on the analogy between single vortex dynamics and charged particle dynamics see Supplementary Note 1).

In superfluid ^4^He, the vortex motion is parallel to the superfluid velocity at temperatures much lower than *T*_c_^14^, whereas observation of the vortex flow anti-parallel to the current in superconductivity has been hindered by the lack of suitable materials. The recently established gate-controlled BCS-BEC crossover is a promising candidate system to investigate the anomalous vortex flow ever closer to the BEC regime. To probe the anomalous vortex motion, we focus on the Hall effect of vortices and its evolution with the carrier density.

The vortex Hall effect (VHE) is a unique transport phenomenon of superconductors, where the collective dynamics of vortices and associated magnetic fluxes produces the Hall voltage^15,16^. The VHE has been recognized since the middle of the last century in several metallic superconductors^17^ and was most intensively investigated in high *T*_c_ cuprates^15,18–22^. Though the potential importance of the BCS-BEC crossover has been discussed for cuprates^1,23^, its impact on the Hall effect^24^ and vortex dynamics remains elusive due to the orders competing with superconductivity such as antiferromagnetism and charge density wave^25^. In particular, it is still unknown how the temperature-dependent sign change of the VHE, which is called Hall anomaly^15^, is related to the BCS-BEC crossover. In the gate-controlled Li_*x*_ZrNCl system, where no competing orders have been observed, intact effects of the BCS-BEC crossover on the VHE and vortex dynamics are expected to be unveiled by transport measurements combined with systematic control of *Δ*/*E*_F_. Even comparing with highly controllable ultracold atomic gases, where transport measurements are developing^26,27^ but still challenging^28^, Li_*x*_ZrNCl is a suitable system to systematically study the vortex dynamics in the BCS-BEC crossover.

Here we report the evolution of the VHE in the BCS-BEC crossover regime via gate-controlled superconductivity of Li_*x*_ZrNCl. We experimentally found that the Hall angle, related to vortex motion, increases with decreasing Li content *x* or electron density towards the crossover regime. Using the time-dependent Ginzburg-Landau (TDGL) model, we successfully explained the temperature dependence of the transverse resistivity and the *x* dependence of the Hall angle in a qualitative manner. The combination of experiments and theory enables us to present a comprehensive picture of vortex dynamics along the BCS-BEC crossover. Leaving the experimental region in the crossover, the expected vortex dynamics in the BEC limit were conjectured, allowing to present a full view on the evolution of vortex dynamics. Therefore, this work shows that the Li_*x*_ZrNCl system allows for an unclouded view on the BCS-BEC crossover rendering it an ideal testbed to benchmark theories on vortex dynamics in the crossover.

## Results

### Experimental observation of the vortex Hall effect in Li_*x*_ZrNCl

Figure 1c, d depict side and top view, respectively, of the crystal structure of the Li-intercalated ZrNCl system (Li_*x*_ZrNCl). The host ZrNCl is a van der Waals layered material with a double honeycomb lattice composed of Zr and N, forming a band insulator. Once Li is intercalated in the van der Waals gap, one electron per Li is introduced to the ZrN conduction layer, and the system exhibits superconductivity^29^. The Li concentration *x* corresponds to the carrier density. Figure 1e displays a schematic band structure of Li_*x*_ZrNCl. Electrons are introduced into the parabolic conduction bands, which are located at the **K** and **K’** points at the corners of the hexagonal Brillouin zone. The electronic phase diagram in the temperature *T* and electron density *x* plane is shown in Fig. 1f. The pseudo-gap state appears at rather high temperature T^*^, and the superconducting transition temperature *T*_c_ exhibits a maximum at *x*~0.01, below which *T*_c_ is well scaled as *T*_c_/*T*_F_~1/8. Furthermore, the ratio of superconducting gap *Δ* to Fermi energy *E*_F_ increases with decreasing *x*^9^. The determination of *Δ* and *E*_F_, which are not specific to superconductor and cold atom systems, enables us to construct a unified experimental phase diagram of the BCS-BEC crossover (Supplementary Note 2 and Supplementary Fig. 1). This encourages us to investigate the nature of BEC superconductivity by extrapolating the trend from the BCS side.

To navigate the single-crystal ZrNCl within the BCS-BEC crossover, an intercalation-only device was employed as previously reported^9,30^. Several doping levels were achieved (Fig. 1f, Supplementary Note 3, and Supplementary Fig. 2). The *T*_c_ data obtained in the present experiment (dark blue) agrees well with the previous results. This phase diagram allows us to correlate observed transport phenomena with the position in the crossover.

**Fig. 2:**
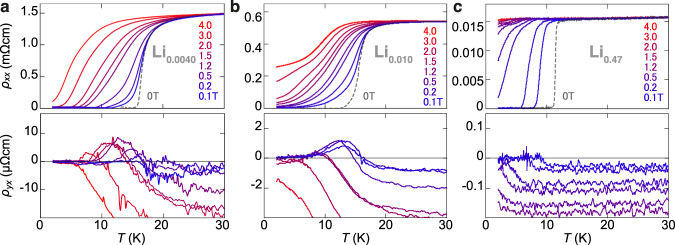
Transport properties at different doping levels *x*. Temperature dependence of longitudinal *ρ*_*xx*_ (top) and transverse *ρ*_*yx*_ (bottom) resistivity at varying out-of-plane fields for *x* = 0.0040 **a**, *x* = 0.010 **b**, and *x* = 0.47 **c**. The vortex Hall effect with sign reversal is clearly observed in **a** and **b**, whereas it is almost indiscernible in (**c**). The magnitude of *ρ*_*yx*_ decreases with increasing *x*.

A detailed look at the transport properties is given with *x* = 0.0040, 0.010, and 0.47 in Fig. 2, which shows the longitudinal and transverse resistivities versus temperature at varying out-of-plane magnetic fields. For the lowest doping, *x* = 0.0040 (Fig. 2a), superconductivity (*T*_c _= 16.8 K) persists even at elevated magnetic fields, and the upper critical field *B*_c2_ reaches 5.9 T (Supplementary Note 4 and Supplementary Fig. 3). No quantum metallic states are visible unlike the electrostatically induced monolayer or bilayer superconductivity^31^. This discrepancy is possibly because the present system is multilayered, similarly to the bulk cuprates. The transverse resistivity *ρ*_*yx*_ (bottom) exhibits anomalous behavior. For *T* > *T*_c_, the Hall signal is negative (*ρ*_*yx*_ < 0), as expected for the n-type transport in electron-doped Li_*x*_ZrNCl. For *T* ~ *T*_c_, however, the transverse resistivity starts to change its sign to *ρ*_*yx*_ > 0 and forms a peak with reducing *T*, which is reminiscent of the Hall anomaly^15^. For medium doping, *x* = 0.010 (Fig. 2b), superconductivity (*T*_c_ = 15.9 K) is less persistent at elevated fields (*B*_c2_ = 5.0 T), and the Hall anomaly is observed though the magnitude is reduced. For high doping, *x* = 0.47 (Fig. 2c), the system shows a sharp transition (*T*_c_ = 11.4 K), a much lower critical field (*B*_c2_ = 0.8 T), and an extremely small VHE. It is important to note that the cleanness, i.e., the ratio of the mean free path over the coherence length has been observed to play a crucial role in the appearance of the Hall anomaly as discussed previously^15^. The doping dependence of the cleanness is shown in Supplementary Fig. 4, to exclude that the observed enhancement of the Hall anomaly versus doping is largely due to a modulation of the cleanness (see Supplementary Note 5 and Supplementary Table 1 for experimentally obtained parameters, including the mean free path and the coherence length).

**Fig. 3:**
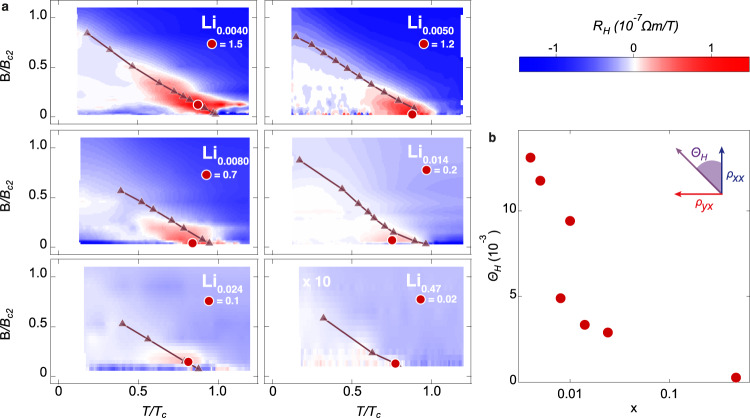
Mapping the vortex Hall effect at different doping levels. **a** Color mapping of the Hall coefficient *R*_H_ on the B-*T* phase diagram for different doping levels. Horizontal and vertical axes are temperature normalized by *T*_c_ and magnetic field normalized by upper critical field *B*_c2_ at zero temperature, respectively. The VHE is visible in the red regions with a sign change of *R*_H_. The areas of VHE are increasing with decreased doping. At the highest doping (*x* = 0.47), the VHE is not visible anymore. Lines and markers show the *T*_c_ (*B*_c2_) superconductivity boundary determined by the half resistance. Red circles mark the maximum *R*_H_ values. **b** Doping dependence of Hall angle *Θ*_H_. With decreasing doping, *Θ*_H_ is enhanced monotonically. In the high doping BCS regime, *Θ*_H_ almost vanishes.

The doping dependence of the VHE can be visualized by mapping the Hall coefficient *R*_H_ (= *ρ*_*yx*_/B) at different doping levels as a function of temperature and field (Fig. 3a). Blue and red areas correspond to the negative and positive *R*_H_, respectively. The red color highlights the enhanced VHE, which appears in the low-field regime around the *B*_c2_ curve, where vortices are highly mobile. Here, the *B*_c2_ curve for each carrier density is determined by the half resistance temperature. These features strongly support that the observed Hall anomalies indeed reflect the VHE. At *x* = 0.47, the VHE almost vanishes. Overall, it is evident that the magnitude and area of the VHE are increasing with decreasing doping. The maximum Hall angle *Θ*_H_, defined at the dark red circles in each phase diagram (Fig. 3a), is summarized as a function of *x* in Fig. 3b. Though the temperature and field, at which *Θ*_H_ is defined (Fig. 3a), vary from sample to sample, *Θ*_H_ characterizes the strength of the VHE at each doping level. The observed *x* dependence of *Θ*_H_ unambiguously implies that the VHE is enhanced by reducing the carrier density, i.e., approaching the BCS-BEC crossover from the BCS side.

### Theoretical modeling of the experimentally observed vortex Hall effect

To elucidate the evolution of vortex dynamics towards the BCS-BEC crossover, observed as the enhanced VHE, we calculated *ρ*_*xx*_ and *ρ*_*yx*_ using the 2D TDGL model (Eq. (1) in Methods) with the gauge invariance^32,33^. Since the sign change of *Θ*_H_ was observed both above and below the *B*_c2_ curve (Fig. 3a), we focused on the dynamics in the vortex liquid state^34^, where many interacting vortices are fluctuating in time and space^16^. Using a few phenomenological parameters and the *E*_F_ dependence of the gap-opening temperature T^*^ (Supplementary Fig. 5), which has been measured previously^9^, we obtained the longitudinal and transverse resistivities near the *B*_c2_ curve (Methods and Supplementary Note 6–9). We assumed a single conduction band to extract a general understanding of vortex dynamics across the BCS-BEC crossover, as independent of material details as possible.

**Fig. 4:**
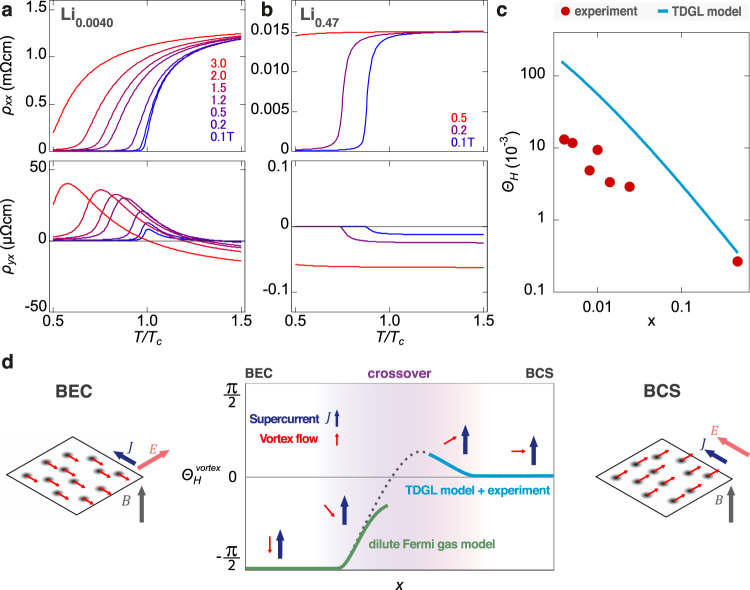
Theoretical simulations of VHE by the TDGL framework. Temperature dependence of the longitudinal resistivity *ρ*_*xx*_ (top) and the transverse resistivity *ρ*_*yx*_ (bottom) at varying out-of-plane fields for the selected doping levels *x* = 0.0040 **a** and 0.47 **b**. **c** Comparison of the theoretical and experimental doping dependence of the Hall angle *Θ*_H_. The experimental data are taken from Fig. 3, whereas the theoretical values are obtained for lower temperatures, where the normal-state contribution is negligible (Supplementary Note 7). **d** Evolution of the vortex Hall angle *Θ*_H_^vortex^ across the BCS-BEC crossover conjectured from the present study. The light blue curve is obtained from the experiment and TDGL model, while the light green curve is obtained theoretically using the diluted Fermi gas model. The dotted line is the interpolation between these two regimes. The blue and red arrows in the inset show the supercurrent and vortex flow, respectively. Schematics in both sides illustrate these relative motions for a set of vortices at the BEC (left) and BCS (right) limits. Here **J**, **E** and **B** are the supercurrent flow, electric field induced by vortex flow, and external magnetic field, respectively. The present experiment is made around the border of the BCS and crossover regimes. It is important to note, the green curve in the BEC regime is a theoretical expectation via the dilute Fermi gas model and is beyond the experimentally supported region of the crossover and BCS regime.

Figure 4a, b shows the obtained temperature dependence of *ρ*_*xx*_ (top) and *ρ*_*yx*_ (bottom) for *x* = 0.0040 and 0.47, respectively. As shown in these figures, we find a qualitative agreement with the experimentally observed temperature and field dependence of *ρ*_*xx*_ and *ρ*_*yx*_, especially the sign change and positive peak of *ρ*_*yx*_ for *x* = 0.0040 (Fig. 2a). This agreement indicates that the peak of *ρ*_*yx*_ occurs in the vortex liquid state, without considering pinning effects. The disappearance of the clear peak in *ρ*_*yx*_ for increased *x* to 0.47 (Fig. 4b) also agrees well with the experiment (Fig. 2c). We have confirmed that these agreements can be obtained even if we change a parameter that is not determined from the experiments (Supplementary Figs. 6 and 7). There are still some discrepancies between experiment and theory. For instance, in the low-temperature high-field region for *x* = 0.0040, the VHE is suppressed in the experiment (Fig. 2a) but still visible in the theory (Fig. 4a). The absence of *ρ*_*yx*_ despite the finite *ρ*_*xx*_ is reminiscent of the quantum vortex liquid state in 2D superconductors^35^. In this state, the vortex is in the liquid state by quantum fluctuation rather than thermal fluctuation^36,37^. This quantum vortex liquid state is left to be confirmed in future studies. In particular, the development of a TDGL model including quantum fluctuations is highly anticipated.

Figure 4c compares the theoretically obtained Hall angle (Methods and Supplementary Note 7) and the experimentally observed *Θ*_H_ (Fig. 3b), which show similar trends of enhancement towards the BCS-BEC crossover. Strictly speaking, the experimental *Θ*_H_ was measured at the peak position of *R*_H_ (Fig. 3a), while the theoretical Hall angle was determined at low temperatures where the vortex contribution to the conductivity is dominant (Supplementary Note 7). Nevertheless, the similar trends in the theory and experiment suggest that the TDGL model successfully captures the evolution of the vortex dynamics and the resulting VHE towards the BCS-BEC crossover. The quantitative difference between the theory and experiment might be attributed to theoretical overestimation of the fluctuation contribution at high fields (see Supplementary Note 9).

The key to the above agreement is that the sign of the Hall angle in the vortex state is opposite to the sign of the *E*_F_ derivative of the mean-field critical temperature in the TDGL model^33^ (Eq. (2) in Methods). In the BCS-BEC crossover, the mean-field critical temperature, which represents the pairing temperature^2,3,23^, should correspond to the gap-opening temperature T^*^ rather than the observed critical temperature *T*_c_. Hence, the sign of the vortex Hall angle, and thereby the occurrence of the Hall anomaly, is determined from that of dT^*^/d*E*_F_, which generally depends on the microscopic details of the system. Since dT^*^/d*E*_F_ < 0 in Li_*x*_ZrNCl (Supplementary Fig. 5), the Hall anomaly observed as the sign change of *Θ*_H_ from negative to positive upon cooling towards the vortex state was theoretically reproduced. This situation (dT^*^/d*E*_F_ < 0) can be microscopically derived from a tight binding model with a finite-range attractive interaction^38^. The simple material nature of Li_*x*_ZrNCl seems important for the applicability of the TDGL model because, in many cuprate superconductors, the simple TDGL model is not applied possibly due to several complex features including the *d*-wave superconductivity^19^ or competing orders such as antiferromagnetism and charge density wave^25^.

## Discussion

The simple material nature of Li_*x*_ZrNCl motivated us to conjecture the evolution of vortex dynamics beyond the experimentally established region in the crossover (Fig. 4c, d light blue curve) towards the BEC limit (Fig. 4d green curve). This way, Fig. 4d displays the expected global evolution of the Hall angle ${\varTheta }_{{{{{{\rm{H}}}}}}}^{{{{{{\rm{vortex}}}}}}}$ in the vortex state with changing the doping level *x* from the BEC to the BCS limit, accompanied with schematics of vortex motions in the two limits. Here we assumed that the vortex state stays liquid over all regions. In the BCS limit, the vortex flow (thin red arrow) is perpendicular to the supercurrent (blue arrow), indicating ${\varTheta }_{{{{{{\rm{H}}}}}}}^{{{{{{\rm{vortex}}}}}}}=0$. The experimentally observed region (light blue line) spans from the BCS to the crossover regime, where the tilting of the vortex motion increases ${\varTheta }_{{{{{{\rm{H}}}}}}}$ as *x* is reduced. This behavior is supported by the theoretical expression of ${\varTheta }_{{{{{{\rm{H}}}}}}}^{{{{{{\rm{vortex}}}}}}}$, i.e., ${{\tan }}{\varTheta }_{{{{{{\rm{H}}}}}}}^{{{{{{\rm{vortex}}}}}}}=\lambda /\gamma \sim O({T}^{*}/{E}_{{{{{{\rm{F}}}}}}})$ (Eq. (2) in Methods and Supplementary Note 7). Here, the coefficients *γ* and *λ* represent typical times for dissipative damping and non-dissipative propagation, respectively. In other words, this equation relates the vortex Hall angle to the relative importance of dissipation ($\gamma /\lambda$): the vortex Hall angle is becoming smaller as the dissipation is growing larger. Thus, in the BCS region, where ${T}^{*}/{E}_{{{{{{\rm{F}}}}}}}\simeq 0$, the dissipation is dominant ($\lambda /\gamma \simeq 0$) and the vortex Hall angle is almost zero. Furthermore, this relation suggests that $\left|{\varTheta }_{{{{{{\rm{H}}}}}}}^{{{{{{\rm{vortex}}}}}}}\right|$ should increase as ${T}^{*}/{E}_{{{{{{\rm{F}}}}}}}$ increases towards the crossover region^9^. In the BEC limit as the low-doping limit, on the other hand, vortices will move anti-parallel to the supercurrent due to the dissipationless vortex core (Fig. 1a), leading to a large negative ${\varTheta }_{{{{{{\rm{H}}}}}}}^{{{{{{\rm{vortex}}}}}}}$. This picture in the dilute limit is also supported by the TDGL model derived from the dilute Fermi gas model with attractive interaction (Supplementary Note 11), which gives vanishing dissipation^23^ ($\gamma=0$ in Eq. (1) in Methods) and accordingly ${\varTheta }_{{{{{{\rm{H}}}}}}}^{{{{{{\rm{vortex}}}}}}}=-\pi /2$ (green line in Fig. 4d). Thus, if one can further reduce *x* from the experimentally observed region, ${\varTheta }_{{{{{{\rm{H}}}}}}}^{{{{{{\rm{vortex}}}}}}}$ is expected to form a positive peak, change its sign, and eventually approach $-\pi /2$ (black dotted line in Fig. 4d). Overall, through the BCS-BEC crossover in Li_*x*_ZrNCl, the vortex dynamics will non-monotonically evolve from dissipative motion perpendicular to the supercurrent into dissipationless motion anti-parallel to the supercurrent.

Figure 4d also provides a comprehensive view of the Hall anomaly throughout the BCS-BEC crossover, corroborated by the TDGL theory. Since ${\varTheta }_{{{{{{\rm{H}}}}}}}$ is negative (i.e., ${\sigma }_{{yx}} \, > \, 0$, see Supplementary Table 2) in the high-temperature normal state of Li_*x*_ZrNCl, the Hall anomaly, i.e., the sign reversal of ${\varTheta }_{{{{{{\rm{H}}}}}}}$ on lowering the temperature (Figs. 2 and 4a), is only expected in the intermediate BCS-BEC crossover regime where ${\varTheta }_{{{{{{\rm{H}}}}}}}^{{{{{{\rm{vortex}}}}}}}$ is positive due to the negative sign of d*T* ^*^/d*E*_F_. (Fig. 4d).

In conclusion, we have systematically investigated the vortex Hall effect in the 2D BCS-BEC crossover using the recently established gate-controlled Li_*x*_ZrNCl system on the experimental side. We experimentally observed the enhancement of the Hall angle with decreasing doping within the crossover. Combined with the TDGL theory, we successfully reproduced this behavior qualitatively, and therefore present the experimentally supported evolution of vortex dynamics within the crossover. This model also allowed to conjecture the expected vortex dynamics beyond the experimental region, towards the BEC limit, unlocking a comprehensive picture of vortex dynamics throughout and beyond the BCS-BEC crossover. The simple band structure of Li_*x*_ZrNCl with absence of competing orders should be crucial to establish a clear understanding of the VHE in many superconductors including cuprates, which has been left unsolved for a long time. The presented density-controlled BCS-BEC crossover has the potential to serve as an essential platform for material exploration and investigations of the unexplored physics of superconductivity in the BEC limit, as showcased herein with the open question of vortex dynamics in the BEC limit left to be tackled experimentally.

## Methods

### Device fabrication

Bulk ZrNCl, prepared by a chemical vapor transport method^39^, was exfoliated onto SiO_2_/Si substrates using the scotch tape technique. The obtained single-crystalline thin flakes exhibited a thickness of ca. 10–40 nm, as determined by atomic force microscopy in tapping mode. Using electron-beam lithography (EBL) with polymethylmethacrylate (PMMA) as positive resist, Au (90 nm)/Cr (7 nm) electrodes were patterned onto a selected thin-flake in a Hall bar setup including a co-planar gate electrode in proximity. In a second EBL step only the edges of the ZrNCl thin flakes and the gate pad were developed, leaving the Hall bar electrodes and the channel region covered with PMMA, to allow for intercalation-only operation of the device^9,30^. For the electrolyte, LiClO_4_ (Sigma Aldrich) was dissolved in polyethylene glycol (PEG, *M*_w _= 600, Wako) at a Li:O (PEG) ratio of 1:20 and stored at 80 °C under vacuum. A drop of electrolyte was placed onto the device to cover both the exposed flake edges and the gate pad. A small cover glass slip was placed on top to evenly spread the electrolyte. The device was transferred into a quantum design physical property measurement system (PPMS) equipped with a rotator probe and high vacuum was applied (<10^−4^ Torr) at 330 K for at least 1 h before measurement.

### Transport measurements

The temperature-dependent resistance of the device at varying magnetic fields was measured in standard four-probe geometry using the PPMS combined with lock-in amplifiers (Stanford Research Systems Model SR830 DSP and Signal Recovery Model 5210) to measure current and voltage. A Keithley 2400 SMU was used to apply the gate voltage at 330 K and high vacuum. The temperature was lowered to 150 K to freeze the PEG-based electrolyte (*T*_m _= 288 K), the chamber was purged with He and the Hall coefficients were measured.

### TDGL model

Assuming that T^*^ corresponds to the mean-field critical temperature, we used the phenomenological two-dimensional (2D) time-dependent Ginzburg-Landau (TDGL) model:

1 $$\left(\gamma+{{{{{\rm{i}}}}}}\lambda \right)\frac{\partial }{\partial t}\varDelta \left({{{{{\bf{r}}}}}},\;t\right)=-\left[\frac{T-{T}^{*}}{{T}^{*}}+b{\left|\varDelta \left({{{{{\bf{r}}}}}},\;t\right)\right|}^{2}-{\xi }^{2}{\left(\nabla+{{{{{\rm{i}}}}}}\frac{2\pi }{{\phi }_{0}}{{{{{\bf{A}}}}}}\left({{{{{\bf{r}}}}}}\right)\right)}^{2}\right]\varDelta \left({{{{{\bf{r}}}}}},\;t\right),$$

where *b* is a phenomenological parameter, ${\phi }_{0}$ is the flux quantum, *ξ* [$={\left({\phi }_{0}/2\pi {B}_{{{{{{\rm{c}}}}}}2}\right)}^{1/2}$] is the coherence length, and *∆*(**r**, *t*) is the superconducting order parameter varying in space and time. For the left-hand side, we simply used the relaxation time derived for the BCS regime^40^, *γ* = π/8 T^*^, and took *λ* = –(1/2 T^*^) ∂T^*^/∂*E*_F_, according to the gauge invariance^32,33^. For the expression of *λ*, we used the mean-field critical temperature T^*^ instead of the observed transition temperature *T*_c_ since the derivation in Aronov et al.^33^ is based only on the linear terms in the TDGL model (1). We also set the chemical potential $\mu$ to ${E}_{{{{{{\rm{F}}}}}}}$ in the expression of $\lambda$ because $\partial {T}^{*}/\partial \mu$ presumably takes a value close to $\partial {T}^{*}/\partial {E}_{{{{{{\rm{F}}}}}}}$ in the experimentally accessible BCS-BEC crossover region; considering the 2D Fermi gas model as a reference^10^, we obtain $(\partial {T}^{*}/\partial \mu )/(\partial {T}^{*}/\partial {E}_{{{{{{\rm{F}}}}}}})\simeq 0.99$ even when the zero-temperature superconducting gap ${\Delta }_{0}$ is as large as ${\Delta }_{0}=0.4{E}_{{{{{{\rm{F}}}}}}}$, which is a typical value observed in Li_x_ZrNCl^9^ (Supplementary Note 10 and Supplementary Fig. 8). We set the vector potential as ${{{{{\bf{A}}}}}}\left({{{{{\bf{r}}}}}}\right)={{{Bx}}}\hat{{{y}}}$ in the Landau gauge, which is fixed so that the scalar potential is zero.

### Calculation of longitudinal and transverse conductivities

Based on the TDGL model, after renormalizing the transition temperature from T^*^ to *T*_c_ (Supplementary Note 6), we applied the linear response theory within the Hartree approximation to obtain the temperature and field dependence of the longitudinal and transverse conductivities (*σ*^V^_*xx*_ and *σ*^V^_*yx*_) for the vortex liquid state^34^ (Supplementary Note 7). To calculate the resistivities (*ρ*_*xx*_ and *ρ*_*yx*_), we introduced the normal-state longitudinal and transverse conductivities (*σ*^N^_*xx*_ and *σ*^N^_*yx*_), which were obtained from the experimental data (Supplementary Table 2). For a phenomenological parameter *β* (∝ *b*), which represents the fluctuation interaction strength, we typically took *β* = 10^−4^–10^−3^, a slight change of which did not affect the qualitative behavior of *ρ*_*xx*_ or *ρ*_*yx*_ (Supplementary Note 9). In Fig. 4a, b, we show the obtained *ρ*_*xx*_ = (*σ*^N^_*xx*_ + *σ*^V^_*xx*_)/[(*σ*^N^_*xx*_ + *σ*^V^_*xx*_)^2^ + (*σ*^N^_*yx*_ + *σ*^V^_*yx*_)^2^] and *ρ*_*yx*_ = –(*σ*^N^_*yx*_ + *σ*^V^_*yx*_)/[(*σ*^N^_*xx*_ + *σ*^V^_*xx*_)^2^ + (*σ*^N^_*yx*_ + *σ*^V^_*yx*_)^2^]. The Hall angle for low enough temperatures can be obtained as (Supplementary Note 7)

2 $${\varTheta }_{{{{{{\rm{H}}}}}}}^{{{{{{\rm{vortex}}}}}}}=-{{\arctan }}\left(\frac{4}{\pi }\frac{\partial {T}^{*}}{\partial {E}_{F}}\right),$$

which is plotted in Fig. 4c.

## Supplementary information

Supplementary Information

## Data Availability

The data that support the plots and other findings of this study are available from the corresponding author upon reasonable request.
