# Supplementary material for: Vortex dynamics in the two-dimensional BCS-BEC crossover
Source: Nat Commun. 2022 Nov 16;13:6986. doi: 10.1038/s41467-022-34756-x (PMC9669018; doi:10.1038/s41467-022-34756-x)
Supplement: Supplementary file 1 — Supplementary Information [file 41467_2022_34756_MOESM1_ESM.pdf]

Supplementary information:
Vortex dynamics in the two-dimensional BCS-BEC crossover

Max Heyl[1,2†], Kyosuke Adachi[3,4†], Yuki M. Itahashi[1], Yuji Nakagawa[1], Yuichi Kasahara[5], Emil J. W. List-Kratochvil[2,6], Yusuke Kato[7], and Yoshihiro Iwasa[1,8*]

Supplementary Note 1: Analogical explanation of vortex dynamics in BCS and BEC limits using charged particle motion

For understanding of the vortex motion both in the BCS and BEC regimes, we compare the dynamics of a single vortex with that of a charged particle. In the presence of a transport current $\boldsymbol{J}_{\rm tr}$, which is perpendicular to the direction of a magnetic field $\boldsymbol{B}$, a single vortex is subject to the driving force given as

$$\boldsymbol{F}_{\rm d} = \boldsymbol{J}_{\rm tr} \times \boldsymbol{\phi}_0, \tag{S1}$$

where the vector $\boldsymbol{\phi}_0$ is parallel to $\boldsymbol{B}$ and has the modulus $|\boldsymbol{\phi}_0| = \phi_0 = h/(2|e|)$ (flux quantum). In addition to $\boldsymbol{F}_{\rm d}$, the vortex also feels the force due to environments such as interactions with impurities, imperfections, or phonons. Assuming that the vortex moves with a velocity $\boldsymbol{v}_{\rm v}$, we can use two independent vectors $\boldsymbol{v}_{\rm v}$ and $\boldsymbol{v}_{\rm v} \times (\boldsymbol{\phi}_0/\phi_0)$ to express the environmental force $\boldsymbol{F}_{\rm env}$:

$$\boldsymbol{F}_{\rm env} = -\eta \boldsymbol{v}_{\rm v} + \eta' \boldsymbol{v}_{\rm v} \times (\boldsymbol{\phi}_0/\phi_0), \tag{S2}$$

where η (> 0) and η' are transport coefficients for the vortex motion. In Eq. (S2), $-\eta \boldsymbol{v}_{\rm v}$ represents a dissipative force since its work is negative $(-\eta \boldsymbol{v}_{\rm v} \cdot \boldsymbol{v}_{\rm v} < 0)$, while $\eta' \boldsymbol{v}_{\rm v} \times (\boldsymbol{\phi}_0/\phi_0)$ is non-dissipative since its work is always zero $[(\eta' \boldsymbol{v}_{\rm v} \times (\boldsymbol{\phi}_0/\phi_0)) \cdot \boldsymbol{v}_{\rm v} = 0]$.

The forces $\boldsymbol{F}_{\rm d}$ and $\boldsymbol{F}_{\rm env}$ are balanced as

$$\boldsymbol{F}_{\rm d} + \boldsymbol{F}_{\rm env} = \boldsymbol{0} \tag{S3}$$

in a steady flow of a single vortex. In the BCS regime, since the dissipative force dominates the non-dissipative force due to the quasi-continuous spectrum in the vortex core (Fig. 1b in the main text), the force balance relation (S3) leads to

$$\boldsymbol{J}_{\rm tr} \times \boldsymbol{\phi}_0 - \eta \boldsymbol{v}_{\rm v} \approx \boldsymbol{0}, \tag{S4}$$

which indicates that the vortex moves perpendicular to the transport current $\boldsymbol{J}_{\rm tr}$. In the BEC regime, since the spectrum is quantized in the vortex and is gapful outside the core (Fig. 1a in the main text), there is no dissipation and the force balance relation (S3) reduces to

$$\boldsymbol{J}_{\rm tr} \times \boldsymbol{\phi}_0 + \eta' \boldsymbol{v}_{\rm v} \times (\boldsymbol{\phi}_0/\phi_0) \approx \boldsymbol{0}, \tag{S5}$$

which yields $\boldsymbol{v}_{\rm v} \approx -(\phi_0/\eta')\boldsymbol{J}_{\rm tr}$, i.e. the vortex motion is anti-parallel to $\boldsymbol{J}_{\rm tr}$.

The force balance relation (S3),

$$\boldsymbol{F}_{\rm d} + \boldsymbol{F}_{\rm env} = \boldsymbol{J}_{\rm tr} \times \boldsymbol{\phi}_0 - \eta \boldsymbol{v}_{\rm v} + \eta' \boldsymbol{v}_{\rm v} \times (\boldsymbol{\phi}_0/\phi_0) = \boldsymbol{0}, \tag{S6}$$

for the vortex motion is less intuitive and thus it could be helpful to rewrite Eq. (S6) in a dual picture so that an analogy with the motion of a charged particle is manifest. Putting $\boldsymbol{\phi}_0 = q\phi_0 \boldsymbol{e}_z$ and $\boldsymbol{J}_{\rm tr} \times \boldsymbol{\phi}_0 =: q\boldsymbol{E}'$ with $q = \pm 1$, Eq. (S6) becomes

$$q\boldsymbol{E}' = \eta \boldsymbol{v}_{\rm v} - q\boldsymbol{v}_{\rm v} \times (\eta' \boldsymbol{e}_z). \tag{S7}$$

We see that Eq. (S7) has the same form as the force balance relation of a charge q in the presence of an "electric field" $\boldsymbol{E}'$ and a "magnetic field" $\boldsymbol{B}' = \eta' \boldsymbol{e}_z$. When $\eta \ll |\eta'|$, which corresponds to the BEC regime, Eq. (S7) describes the motion of a charged particle under a strong "magnetic field" $\boldsymbol{B}'$ and thus the motion is almost perpendicular to $q\boldsymbol{E}'$ and $\boldsymbol{v}_{\rm v}$ is anti-parallel to $\boldsymbol{J}_{\rm tr}$. When $\eta \gg |\eta'|$, which corresponds to the BCS regime, Eq. (S7) describes the motion of a charged particle under a weak "magnetic field" $\boldsymbol{B}'$ and thus the motion is almost parallel to $q\boldsymbol{E}'$ and $\boldsymbol{v}_{\rm v}$ is perpendicular to $\boldsymbol{J}_{\rm tr}$. Retaining a small $|\eta'|$, we obtain a small Hall angle, the sign of which depends on that of η', i.e. the direction of $\boldsymbol{B}'$.

Supplementary Note 2: Unified experimental BCS-BEC crossover phase diagram

The phase diagram of superconductors is usually drawn on the T-carrier density plane, whereas, in cold atom systems, the phase diagram is often drawn on the plane of T-$1/k_{\mathrm{F}}a_s$, where k_{F} and a_s denote the Fermi vector and scattering length, respectively, and thus $1/k_{\mathrm{F}}a_s$ represents the normalized interaction strength. Therefore, it has not been possible to directly compare the two BCS-BEC crossover systems yet. Recently, we presented a phase diagram on the T/T_{F}-Δ/E_{F} plane for 2D superconductors [1], which is free from the parameters specific to superconductors. On the other hand, in the 2D ^{6}Li system, the experimental determination of Δ/E_{F} as a function of the interaction strength was recently reported [2]. Combining the phase diagram on the same system published in 2015 [3], we are able to draw a phase diagram on the T/T_{F}-Δ/E_{F} plane for the 2D ^{6}Li system. This allows us to construct a unified experimental phase diagram of the BCS-BEC crossover, which is displayed in Supplementary Fig. 1. Though there remain discrepancies due to the difference in definition of each parameter, the phase diagram shows that the data of Li$_x$ZrNCl and ^{6}Li just overlap with each other and encourages us to consider the BEC limit from the BCS side.

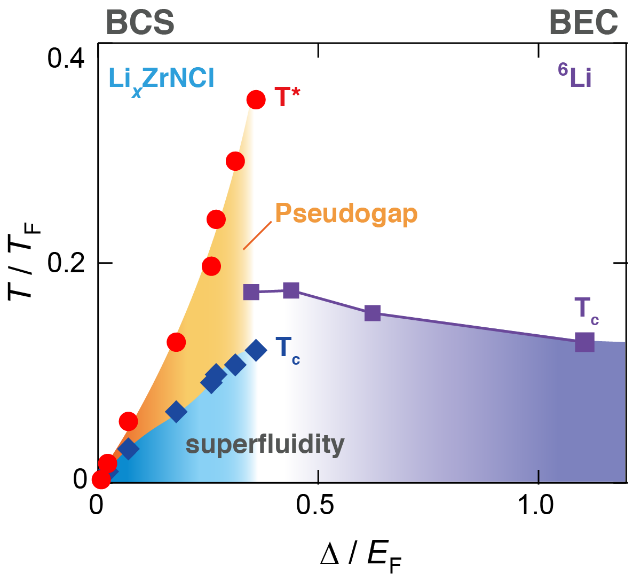

Supplementary Figure 1. **A unified BCS-BEC crossover phase diagram from combined experimental data for cold atom superfluids ^{6}Li and density-controlled superconductors Li$_x$ZrNCl.** Starting from the BCS side, the data points for the 2D superconductor Li$_x$ZrNCl [1], the system studied in this work, are drawn. Here T^* is the gap-opening temperature and T_{c} is the critical temperature. Starting from the BEC side, the purple data points correspond to the cold atom superfluid ^{6}Li, taken from previous reports [2, 3]. Drawing the phase diagram for both systems of superconductivity and cold atom superfluidity on a common scale reveals the achieved overlap of data points in the BCS-BEC crossover. This ultimately motivates the study of BEC superconductivty starting from the BCS regime in Li$_x$ZrNCl.

Supplementary Note 3: Basic transport properties and doping level determination

To determine the doping level, i.e., Li content x, the Hall effect was used. The linear slope of Supplementary Fig. 2a was used to determine x and a systematic dependency of the slope with changing Li ion concentration is apparent. The device operation for intercalation was analogous to previously established work [4]. To compute the Li content, the Hall coefficient at 150 K was measured and we assume that each Li ion supplies one electron to the ZrNCl system. For the determined doping levels, superconductivity was achieved, and we show the longitudinal resistivity as a function of temperature for each in Supplementary Fig. 2b. With decreasing doping level, the critical temperature T_{c} increased from 11.4 K to 16.8 K for $x = 0.47$ and 0.0040 respectively. T_{c} was determined by the temperature at which the resistivity is half of the normal-state value at 30 K. The superconducting transition is sharp for high doping levels but is significantly broadened towards lower doping levels. This may be explained by the enhanced fluctuation strength (β in Supplementary Figs. 6 and 7) towards the BCS-BEC crossover. In addition, the dimensional crossover from an anisotropic 3D superconductor to a 2D superconductor can be relevant since the dimensional crossover occurs around $x \sim 0.1$, as discussed in previous works [1, 4]. In the low doping regime, the transition is better described by the Berezinskii-Kosterlitz-Thouless (BKT) transition using T_{BKT} instead of T_{c}. However, the T_{c} values are sufficiently close to T_{BKT} to lend themselves for our further comparison.

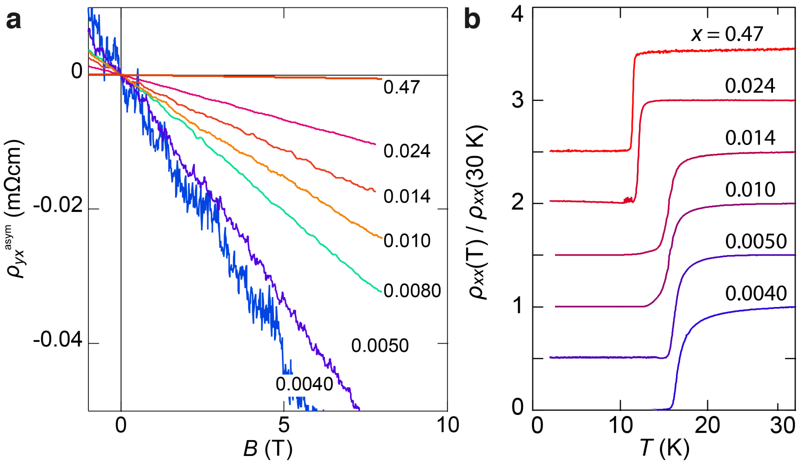

Supplementary Figure 2. **Doping level determination and superconducting transitions.**
(**a**) Anti-symmetrized transverse resistivity as a function of magnetic field measured at 150 K to determine the doping levels as annotated. The slope of the Hall signal is used to calculate x. (**b**) Doping dependence of the superconducting transition. Lower Li contents show higher critical temperatures T_{c} and broadened transitions due to superconducting fluctuation. Resistivity was normalized at 30 K and the curves are each shifted by 0.5.

Supplementary Note 4: Doping dependence of the upper critical field

The out-of-plane upper critical field B_{c2} was determined by measuring the temperature dependence of resistivity at several magnetic fields. For the normal state, resistivity at high temperatures (> 30 K) under the highest applied out-of-plane field of 8.8 T was chosen. The transition point is then defined as the half value of the normal state. These transition points are plotted as a function of temperature for each applied field at several doping levels in Supplementary Fig. 3a. Linear extrapolations to 0 K are plotted, which are used to determine B_{c2} at zero temperature. The enhancement of the upper critical field with decreased doping is evident, as also seen in Supplementary Fig. 3b, where the doping dependence of B_{c2} is shown. By using the Ginzburg-Landau (GL) model, $B_{\mathrm{c2}}(T) = (\phi_0/2\pi\xi^2)(1 - T/T_{\mathrm{c}})$, where ϕ_0 is the flux quantum, one can compute the in-plane coherence length at zero temperature (ξ) by using the slope of the linear $B_{\mathrm{c2}}(T)$ relation. The values for each doping level are documented in Supplementary Table 1. For decreasing doping, ξ is decreasing. This indicates the realization of strongly coupled small Cooper pairs in the low-carrier density regime.

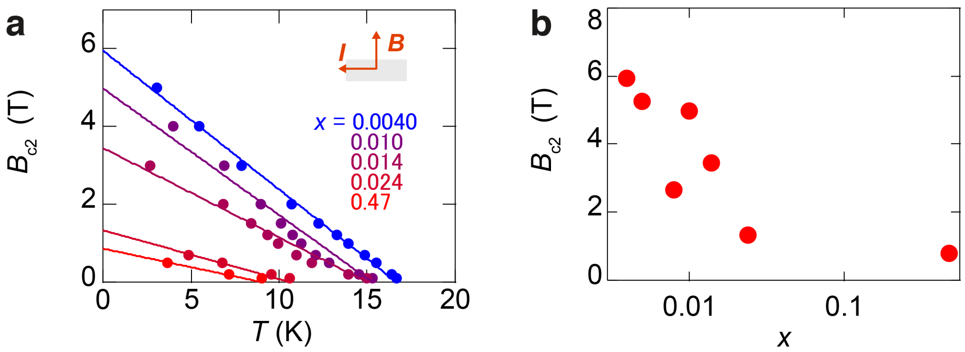

Supplementary Figure 3. **Out-of-plane upper critical fields B_{c2}.**
(**a**) B_{c2} as a function of temperature at several doping levels. Solid lines represent linear extrapolations to 0 K for each doping level. (**b**) Doping dependence of B_{c2}.

Supplementary Note 5: Summary of superconductivity properties at several doping levels

In Supplementary Table 1, the summarized values of the Li content x, carrier density n at 150 K, Fermi energy E_{F}, Fermi wave vector k_{F}, critical temperature T_{c}, out-of-plane upper critical field B_{c2} linearly extrapolated to 0 K, in-plane coherence length ξ at 0 K, Hall mobility μ at 30 K and mean free path ℓ at 30 K for various achieved x in $\mathrm{Li}_x\mathrm{ZrNCl}$ are shown. To calculate E_{F} and k_{F} from n, an ideal parabolic band dispersion in two-dimensions was

employed. $k_{\mathrm{F}} = (4\pi n_{\mathrm{layer}}/ss')^{1/2}$ and $E_{\mathrm{F}} = \hbar^2 k_{\mathrm{F}}^2/2m^*$, with n_{layer} the 2D carrier density per layer, s the spin degree of freedom, s' the valley degree of freedom, $\hbar$ the reduced Planck constant and m^* the effective electron mass. In the case of $\mathrm{Li}_x\mathrm{ZrNCl}$, $s = s' = 2$ and $m^* = 0.9m_0$, as reported before [5]. Here m_0 denotes the free electron mass. The Hall mobility was computed using $\mu = -\sigma_{xx}R_{\mathrm{H}}$, where σ_{xx} is the longitudinal conductivity and R_{H} is the Hall coefficient. The mean free path was computed via $\ell = v_{\mathrm{F}}\mu\, m^* q^{-1}$, where q is the charge and v_{F} is the Fermi velocity computed via $v_{\mathrm{F}} = \hbar k_{\mathrm{F}}/m^*$.

Supplementary Table 1. **Summary of parameters for doping-dependent superconductivity in $\mathrm{Li}_x\mathrm{ZrNCl}$.**

x	$n(\times 10^{20}\ \mathrm{cm}^{-3})$	$E_{\mathrm{F}}(\mathrm{meV})$	$k_{\mathrm{F}}(\mathrm{nm}^{-1})$	$T_{\mathrm{c}}(\mathrm{K})$	$B_{\mathrm{c2}}(\mathrm{T})$	$\xi(\mathrm{nm})$
0.0040	0.718	8.8	0.456	16.8	5.95	7.44
0.0050	0.958	11.7	0.526	16.4	5.26	7.91
0.010	2.02	24.8	0.765	15.9	4.98	8.13
0.014	2.76	33.7	0.893	15.8	3.45	9.77
0.024	4.74	58.0	1.17	12.1	1.33	15.71
0.47	90.48	1107.0	5.11	11.4	0.79	20.47

x	$\mu_{30\mathrm{K}}(\mathrm{cm}^2\mathrm{V}^{-1}\mathrm{s}^{-1})$	$\ell_{30\mathrm{K}}(\mathrm{nm})$
0.0040	68.42	5.79
0.0050	70.13	6.54
0.010	55.03	6.58
0.014	44.54	5.90
0.024	42.52	6.75
0.47	86.28	36.61

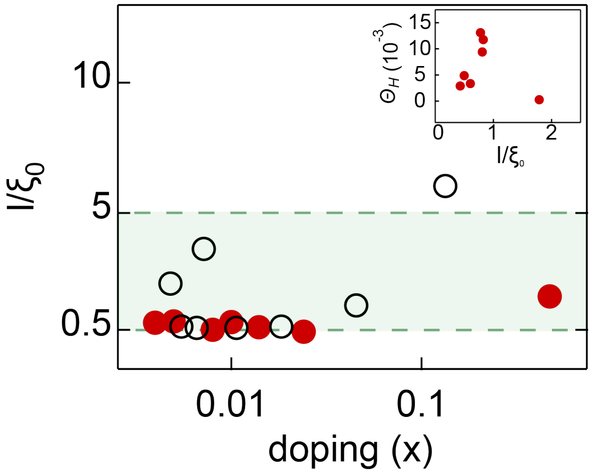

Supplementary Figure 4. **Doping dependence of the ratio of the mean free path l and superconducting coherence length ξ_0.**
Red dots represent the data points from this work while black circles represent the data from the previous work [1]. The green area highlights the moderately clean regime where $0.5 < l/\xi_0 < 5$, above or below which the Hall anomaly is seemingly not observable as described by Hagen et al. [6]. The inset shows the Hall angle dependence on l/ξ_0. Almost no modulation of the l/ξ_0 ratio with doping is observed, which is also reflected in the weak dependence of the Hall angle on this ratio. This concludes that the system stays in the relevant range of l/ξ_0 over the course of this work and the observed trend in the Hall angle vs doping is not dominated by a change of this ratio.

Supplementary Note 6: Theoretical determination of T_{c} by Hartree approximation

As explained in Methods, the time-dependent Ginzuburg-Landau (TDGL) model is given by

$$(\gamma + \mathrm{i}\lambda)\frac{\partial}{\partial t}\Delta(\boldsymbol{r},t) = -\left[\frac{T - T^*}{T^*} + b|\Delta(\boldsymbol{r},t)|^2 - \xi^2\left(\boldsymbol{\nabla} + \mathrm{i}\frac{2\pi}{\phi_0}\boldsymbol{A}(\boldsymbol{r})\right)^2\right]\Delta(\boldsymbol{r},t), \tag{S8}$$

where $\gamma = \pi/8T^*$, $\lambda = -(1/2T^*)\partial T^*/\partial E_{\mathrm{F}}$, $\xi = \sqrt{\phi_0/2\pi B_{\mathrm{c2}}(0)}$, $\boldsymbol{A}(\boldsymbol{r}) = Bx\hat{y}$, and $\Delta(\boldsymbol{r},t)$ is the superconducting order parameter varying in space and time.

We consider the GL Hamiltonian corresponding to Eq. (S8) as

$$H_{\mathrm{GL}} := a \int \mathrm{d}^2\boldsymbol{r} \left[\frac{T-T^*}{T^*}|\Delta(\boldsymbol{r})|^2 + \frac{b}{2}|\Delta(\boldsymbol{r})|^4 + \xi^2 \left| \left(\boldsymbol{\nabla} + \mathrm{i}\frac{2\pi}{\phi_0}\boldsymbol{A}(\boldsymbol{r}) \right) \Delta(\boldsymbol{r}) \right|^2 \right], \tag{S9}$$

where a is another phenomenological parameter. Replacing $|\Delta|^4$ with $2\langle|\Delta|^2\rangle|\Delta|^2$ in Eq. (S9) by the Hartree approximation, we obtain the approximated Hamiltonian

$$H'_{\mathrm{GL}} := a \int \mathrm{d}^2\boldsymbol{r} \left[\epsilon|\Delta(\boldsymbol{r})|^2 + \xi^2 \left| \left(\boldsymbol{\nabla} + \mathrm{i}\frac{2\pi}{\phi_0}\boldsymbol{A}(\boldsymbol{r}) \right) \Delta(\boldsymbol{r}) \right|^2 \right]. \tag{S10}$$

Here, the renormalized mass ϵ satisfies the self-consistent equation:

$$\epsilon = \frac{T-T^*}{T^*} + b\langle|\Delta|^2\rangle, \tag{S11}$$

where $\langle\cdots\rangle$ is the canonical average using the Hamiltonian H'_{GL} and the temperature T. Expanding $\Delta(\boldsymbol{r})$ as $\Delta(\boldsymbol{r}) = \sum_{N,q} c_{Nq}\varphi_{Nq}(\boldsymbol{r})$ with the eigenfunction $\varphi_{Nq}(\boldsymbol{r}) \propto \mathrm{H}_N(x/l+ql)\exp[-(x/l+ql)^2/2 + \mathrm{i}qy]$, we can diagonalize H'_{GL} as $H'_{\mathrm{GL}} = a\sum_{N,q}[\epsilon + (2N+1)h]|c_{Nq}|^2$. Here, N and q are the Landau level index and its degeneracy index, respectively, $H_N(z)$ is the Nth Hermite polynomial, $l := \sqrt{\phi_0/2\pi B}$, and the dimensionless magnetic field is defined as $h := (\xi/l)^2 = B/B_{\mathrm{c2}}(0)$. Then, the self-consistent equation (S11) may be rewritten as

$$\epsilon = \frac{T-T^*}{T^*} + \frac{bTh}{2\pi a\xi^2}\sum_{N=0}^{c/h}\frac{1}{\epsilon + (2N+1)h}, \tag{S12}$$

where c is a cutoff parameter representing the limitation of the gradient expansion in the GL Hamiltonian (S9).

We further rewrite Eq. (S12) as $\epsilon = (T-T^*)/T^* + (bT/4\pi a\xi^2)\{[\sum_{N=0}^{c/h}(N+\epsilon/2h+1/2)^{-1} - \ln(c/h)] + \ln(c/h)\}$. Assuming large enough c/h and taking $c/h\to\infty$ in $[\cdots]$ in the right-hand side, we can obtain

$$\epsilon = \frac{T-T^*}{T^*} + \frac{bT}{4\pi a\xi^2}\left[-\psi\left(\frac{\epsilon+h}{2h}\right) + \ln\frac{c}{h} \right], \tag{S13}$$

where $\psi(z)$ is the digamma function, which satisfies $\psi(z) = \lim_{n\to\infty}[\ln n - \sum_{m=0}^{n}(m+z)^{-1}]$. To express c by the renormalized transition temperature, T_{c}, we temporarily consider $h\to 0$ in Eq. (S13) and use $\psi(z)\to\ln z + O(z^{-1})$ for $z\to\infty$. Then, we can obtain the equation for zero magnetic field as

$$\epsilon = T\left[\frac{1}{T^*} + \frac{b\ln(2c)}{4\pi a\xi^2} \right] - 1 - \frac{bT}{4\pi a\xi^2}\ln\epsilon. \tag{S14}$$

Based on the right-hand side of Eq. (S14), we define the renormalized T_{c} as

$$T_{\mathrm{c}} := \left[\frac{1}{T^*} + \frac{b\ln(2c)}{4\pi a\xi^2} \right]^{-1}. \tag{S15}$$

Note that the spontaneous symmetry breaking does not occur at finite temperatures since we consider a 2D system, and T_{c} characterizes a typical temperature for significant changes in physical quantities such as conductivity.

Using the expression of T_{c} [Eq. (S15)] in Eq. (S13), we finally obtain the equation to determine ϵ as a function of T/T_{c} and h [$= B/B_{\mathrm{c2}}(0)$]:

$$\epsilon = \frac{T-T_{\mathrm{c}}}{T_{\mathrm{c}}} + \beta\frac{T}{T_{\mathrm{c}}}\left[-\psi\left(\frac{\epsilon+h}{2h}\right) - \ln(2h) \right]. \tag{S16}$$

Here, β ($:= bT_{\mathrm{c}}/4\pi a\xi^2$) is the dimensionless fluctuation interaction strength.

Supplementary Note 7: Longitudinal and transverse conductivities

In the following, we derive the electrical conductivity σ^{V}_{xx} and σ^{V}_{yx} due to the vortex liquid using the Hartree approximation [7]. According to the linear response theory [8], we can express σ^{V}_{ab} as

$$\sigma^{\mathrm{V}}_{ab} = \frac{1}{TS}\int_0^\infty \mathrm{d}t \int \mathrm{d}^2\boldsymbol{r} \int \mathrm{d}^2\boldsymbol{r}' \langle j_a(\boldsymbol{r},t)j_b(\boldsymbol{r}',0)\rangle, \tag{S17}$$

where S is the total area of the system, and $\boldsymbol{j}(\boldsymbol{r},t)$ is the local current density defined as

$$\boldsymbol{j}(\boldsymbol{r},t) := \frac{2\pi}{\phi_0} a\xi^2 \left\{ \mathrm{i}(\boldsymbol{\nabla}_1 - \boldsymbol{\nabla}_2) - \frac{2\pi}{\phi_0}[\boldsymbol{A}(\boldsymbol{r}_1) + \boldsymbol{A}(\boldsymbol{r}_2)] \right\} \Delta(\boldsymbol{r}_1,t)\Delta^*(\boldsymbol{r}_2,t)\Big|_{\boldsymbol{r}_1=\boldsymbol{r}_2=\boldsymbol{r}}. \tag{S18}$$

Within the Hartree approximation [7], $\langle\cdots\rangle$ in Eq. (S17) means the canonical average using H'_{GL} [Eq. (S10)] for the initial state. Using ϵ, which satisfies Eq. (S16), the TDGL model (S8) is approximated as

$$(\gamma + \mathrm{i}\lambda)\frac{\partial}{\partial t}\Delta(\boldsymbol{r},t) = -\left[\epsilon - \xi^2\left(\boldsymbol{\nabla} + \mathrm{i}\frac{2\pi}{\phi_0}\boldsymbol{A}(\boldsymbol{r})\right)^2\right]\Delta(\boldsymbol{r},t). \tag{S19}$$

Expanding $\Delta(\boldsymbol{r},t)$ as $\Delta(\boldsymbol{r},t) = \sum_{N,q} c_{Nq}(t)\varphi_{Nq}(\boldsymbol{r})$, we can solve Eq. (S19) as $c_{Nq}(t) = c_{Nq}\exp[-(\epsilon+h+2hN)t/(\gamma+\mathrm{i}\lambda)]$ with $c_{Nq} := c_{Nq}(0)$. Thus, Eq. (S17) leads to

$$\sigma^{\mathrm{V}}_{ab} = \frac{1}{TS}\left(\frac{2\pi}{\phi_0}a\xi^2\right)^2 \sum_{N,N',q,q'} \langle|c_{Nq}|^2\rangle\langle|c_{N'q'}|^2\rangle \int_0^\infty \mathrm{d}t\, \mathrm{e}^{-\Gamma(N,N')t}$$

$$\times \int \mathrm{d}^2\boldsymbol{r} \int \mathrm{d}^2\boldsymbol{r}' [\hat{\boldsymbol{\Pi}}_1 + \hat{\boldsymbol{\Pi}}^*_2]_a [\hat{\boldsymbol{\Pi}}'_1 + \hat{\boldsymbol{\Pi}}'^*_2]_b \varphi_{Nq}(\boldsymbol{r}_1)\varphi^*_{N'q'}(\boldsymbol{r}_2)\varphi_{N'q'}(\boldsymbol{r}'_1)\varphi^*_{Nq}(\boldsymbol{r}'_2)|_{\boldsymbol{r}^{(\prime)}_1=\boldsymbol{r}^{(\prime)}_2=\boldsymbol{r}^{(\prime)}}, \tag{S20}$$

where $\Gamma(N,N') := 2\{\gamma[\epsilon+(N_1+N_2+1)h] - \mathrm{i}\lambda(N_1-N_2)h\}/(\gamma^2+\lambda^2)$, and $\hat{\boldsymbol{\Pi}}^{(\prime)}_i$ $[:= -\mathrm{i}\boldsymbol{\nabla}^{(\prime)}_i + (2\pi/\phi_0)\boldsymbol{A}(\boldsymbol{r}^{(\prime)}_i)]$ is the gauge-invariant momentum operator. Using the ladder operators $\hat{a}_q := (x+ql^2+l^2\partial_x)/\sqrt{2}l$ and $\hat{a}^\dagger_q := (x+ql^2-l^2\partial_x)/\sqrt{2}l$, we can obtain the relations such as $\hat{\Pi}_x\varphi_{Nq}(\boldsymbol{r}) = -(\mathrm{i}/\sqrt{2}l)(\hat{a}_q - \hat{a}^\dagger_q)\varphi_{Nq}(\boldsymbol{r}) = -(\mathrm{i}/\sqrt{2}l)[\sqrt{N}\varphi_{N-1,q}(\boldsymbol{r}) - \sqrt{N+1}\varphi_{N+1,q}(\boldsymbol{r})]$. Performing the time and space integrations and the canonical average in Eq. (S20), we can finally obtain the following formulas:

$$\sigma^{\mathrm{V}}_{xx} = \sigma^{\mathrm{V}}_{yy} = \frac{\gamma T h^2}{\pi}\left(\frac{2\pi}{\phi_0}\right)^2\left(1+\frac{\lambda^2}{\gamma^2}\right)\sum_{N=0}^{c/h}\frac{(N+1)\mu_{N+1/2}}{\mu_N\mu_{N+1}({\mu_{N+1/2}}^2 + \lambda^2h^2/\gamma^2)}, \tag{S21}$$

$$\sigma^{\mathrm{V}}_{yx} = -\sigma^{\mathrm{V}}_{xy} = -\frac{\lambda T h^3}{\pi}\left(\frac{2\pi}{\phi_0}\right)^2\left(1+\frac{\lambda^2}{\gamma^2}\right)\sum_{N=0}^{c/h}\frac{N+1}{\mu_N\mu_{N+1}({\mu_{N+1/2}}^2 + \lambda^2h^2/\gamma^2)}, \tag{S22}$$

where $\mu_N := \epsilon + h + 2Nh$, and a cutoff c is introduced similarly to Eq. (S12).

For $\epsilon + h \ll h$, the $N = 0$ terms are dominant in Eqs. (S21) and (S22), and we obtain the asymptotic expressions as

$$\sigma^{\mathrm{V}}_{xx} = \sigma^{\mathrm{V}}_{yy} \simeq \frac{2\pi\gamma T}{{\phi_0}^2(\epsilon+h)} \qquad (\text{for } \epsilon + h \ll h), \tag{S23}$$

$$\sigma^{\mathrm{V}}_{yx} = -\sigma^{\mathrm{V}}_{xy} \simeq -\frac{2\pi\lambda T}{{\phi_0}^2(\epsilon+h)} \qquad (\text{for } \epsilon + h \ll h). \tag{S24}$$

Thus, for low enough temperatures, where $\epsilon + h \ll h$ and σ^{V}_{ab} dominates over the normal-state conductivity σ^{N}_{ab}, the resistivities are given as $\rho_{xx} := (\sigma^{\mathrm{N}}_{xx} + \sigma^{\mathrm{V}}_{xx})/[(\sigma^{\mathrm{N}}_{xx} + \sigma^{\mathrm{V}}_{xx})^2 + (\sigma^{\mathrm{N}}_{yx} + \sigma^{\mathrm{V}}_{yx})^2] \simeq \sigma^{\mathrm{V}}_{xx}/[(\sigma^{\mathrm{V}}_{xx})^2 + (\sigma^{\mathrm{V}}_{yx})^2]$ and $\rho_{yx} := -(\sigma^{\mathrm{N}}_{yx} + \sigma^{\mathrm{V}}_{yx})/[(\sigma^{\mathrm{N}}_{xx} + \sigma^{\mathrm{V}}_{xx})^2 + (\sigma^{\mathrm{N}}_{yx} + \sigma^{\mathrm{V}}_{yx})^2] \simeq -\sigma^{\mathrm{V}}_{yx}/[(\sigma^{\mathrm{V}}_{xx})^2 + (\sigma^{\mathrm{V}}_{yx})^2]$, and the Hall angle Θ_{H} follows

$$\tan\Theta_{\mathrm{H}} := \rho_{yx}/\rho_{xx} \simeq -\sigma^{\mathrm{V}}_{yx}/\sigma^{\mathrm{V}}_{xx} \simeq \lambda/\gamma. \qquad (\text{for } \epsilon + h \ll h,\ \sigma^{\mathrm{N}}_{xx} \ll \sigma^{\mathrm{V}}_{xx},\ \text{and } |\sigma^{\mathrm{N}}_{yx}| \ll |\sigma^{\mathrm{V}}_{yx}|) \tag{S25}$$

By explicitly setting $\gamma = \pi/8T^*$ and $\lambda = -(\partial T^*/\partial E_{\mathrm{F}})/2T^*$ in (S25), we finally obtain

$$\tan\Theta_{\mathrm{H}} \simeq -(4/\pi)\partial T^*/\partial E_{\mathrm{F}}. \tag{S26}$$

Supplementary Note 8: TDGL equation and Hall conductivity

We discuss the crucial role of λ in the left-hand-side of Eq. (S8). When $\lambda = 0$, Eq. (S8) has a particle-hole symmetry, i.e, When Δ and $\boldsymbol{A}$ satisfy the Eq. (S8) with $\lambda = 0$, $\Delta' = \Delta^*$ and $\boldsymbol{A}' = -\boldsymbol{A}$ do the same equation. Both the current density and electric field change their sign and it thus follows that $\sigma_{xy}(\boldsymbol{B}) = \sigma_{xy}(-\boldsymbol{B})$ under

this transformation for $\boldsymbol{B}$ parallel to z-axis. The Onsager relation $\sigma_{xy}(\boldsymbol{B}) = \sigma_{yx}(-\boldsymbol{B})$ together with the rotational symmetry $\sigma_{xy}(\boldsymbol{B}) = -\sigma_{yx}(\boldsymbol{B})$ in the xy plane yields $\sigma_{xy}(\boldsymbol{B}) = -\sigma_{xy}(-\boldsymbol{B})$. We thus see that $\sigma_{xy}(\boldsymbol{B}) = 0$ when $\lambda = 0$.

As we will confirm in the following calculation, the sign of the λ determines that of the Hall conductivity. Before explicit calculation, we discuss the sign of the Hall conductivity in an intuitive way. For simplicity, we set $\gamma = 0$, which is irrelevant to the sign of the Hall conductivity, then Eq. (S8) reduces to the form of non-linear Schrödinger equation, where λ corresponds to $-m^*\xi^2/\hbar$ with the inertial mass m^* of a Cooper pair. When $\lambda < 0$, the Eq. (S8) with $\gamma = 0$ describes the dynamics of charged condensate with a positive inertial mass and the resultant Hall conductivity with the same sign as the electrons in the normal state. When $\lambda > 0$, on the other hand, the same equation describes the dynamics of charged condensate with a negative mass. In this case, the Hall effect due to motion of condensate has the opposite sign to that in the normal state.

To gain further insight into the dynamics of the condensate, it would be helpful to to rewrite $\Delta(\boldsymbol{r}, t)$ as $|\Delta(\boldsymbol{r}, t)|e^{i\chi(\boldsymbol{r},t)}$ and decompose TDGL equation (S8) multiplied by $e^{-i\chi(\boldsymbol{r},t)}$ into real and imaginary parts

$$\gamma\frac{\partial|\Delta|}{\partial t} = -\frac{1}{2a}\frac{\delta H_{\rm GL}}{\delta|\Delta|} + \lambda|\Delta|\frac{\partial\chi}{\partial t} \tag{S27a}$$

$$\frac{\partial\rho_{\rm s}}{\partial t} + \nabla\cdot\boldsymbol{j}_{\rm s} = -\frac{4\pi\gamma|\Delta|^2}{\phi_0}\frac{\partial\chi}{\partial t}. \tag{S27b}$$

The GL Hamiltonian (S9) is introduced in eq. (S27a), which describes the relaxation dynamics of the condensate. In eq. (S27b), we introduce the notation:

$$\rho_{\rm s} := \frac{2\pi a\lambda|\Delta|^2}{\phi_0}, \quad \boldsymbol{j}_{\rm s} = -2\left(\frac{2\pi|\Delta|\xi}{\phi_0}\right)^2 \underbrace{\left(\boldsymbol{A} + \frac{\phi_0}{2\pi}\nabla\chi\right)}_{=:\boldsymbol{Q}}, \tag{S28}$$

the latter of which is nothing but (S18). We can regard Eq.(S27b) as the equation of continuity of the superfluid component of charge and current with the sink/source term. Conserved is the sum of the superfluid component and normal component of charge density. We thus interpret the righ-hand side of eq. (S27b) as the conversion rate of charge density from the normal to superfluid component and introduce the notation

$$\left[\frac{d\rho_{\rm s}}{dt}\right]_{\rm conv} := -\frac{4\pi\gamma|\Delta|^2}{\phi_0}\frac{\partial\chi}{\partial t}. \tag{S29}$$

In Eq. (S28) for $\lambda < 0$, $\rho_{\rm s}$ is negative and corresponds to the positive electron density. Thus the dynamics of the condensate is similar to the electron motion. In Eq. (S28) for $\lambda > 0$, $\rho_{\rm s}$ is positive and corresponds to the deficit of electron number density. We then expect the dynamics of the condensate is similar to that of holes. Further we can discuss the dynamics of the condensate on the basis of momentum balance relation, which corresponds to the Euler equation (equation of motion) in hydrodynamics

$$\frac{\partial(-\rho_{\rm s}\boldsymbol{Q})}{\partial t} + \boldsymbol{\nabla}\cdot\boldsymbol{\mathcal{P}} = \rho_{\rm s}\boldsymbol{\varepsilon} + \boldsymbol{j}_{\rm s}\times\boldsymbol{h} + 2a\gamma\frac{\partial|\Delta|}{\partial t}\boldsymbol{\nabla}|\Delta| - \left[\frac{d\rho_{\rm s}}{dt}\right]_{\rm conv}\boldsymbol{Q}, \tag{S30}$$

which follows from Eqs. (S27a) and (S27b), and the Ampere-Maxwell equation (see derivation of [9]). Let us see the physical meaning of each term in order to confirm that this equation is really regarded as the momentum balance relation. We start with the right-hand side. Here the electric magnetic fields are denoted by $\boldsymbol{\varepsilon} = -\partial\boldsymbol{A}/\partial t$ and $\boldsymbol{h} = \boldsymbol{\nabla}\times\boldsymbol{A}$. The first two terms in the right-hand side represent the electromagnetic Lorentz force. The third term in the right-hand side is the dissipation force due to the time variation of the modulus of $|\Delta|$ (This mechanism was first pointed out by Tinkham [10]). The last term in the right-hand side in Eq. (S30) is the other dissipation force due to conversion between the superfluid and normal components. This disspative force is caused by the time-variation of the phase of Δ. Thus these two terms show that the vortex motion is the source of the dissipative force. In the left-hand side, $\boldsymbol{\mathcal{P}}$ represents the hydrodynamic momentum flux tensor, which is given in the present case by

$$(\boldsymbol{\mathcal{P}})_{\mu\nu} = -j_{{\rm s},\mu}Q_\nu + 2a\xi^2\partial_\mu|\Delta|\partial_\nu|\Delta| - \delta_{\mu\nu}\left(a\mathcal{F} - \rho_{\rm s}\frac{\phi_0}{2\pi}\frac{\partial\chi}{\partial t}\right) \tag{S31}$$

$$\mathcal{F} = \frac{T-T^*}{T^*}|\Delta|^2 + \frac{b}{2}|\Delta|^4 + \xi^2\left(\boldsymbol{\nabla}|\Delta|\right)^2 + \left(\frac{2\pi\xi}{\phi_0}|\Delta|\boldsymbol{Q}\right)^2. \tag{S32}$$

These expressions in the London limit, where $|\Delta|$ is spatially uniform, reduce to

$$(\boldsymbol{\mathcal{P}})_{\mu\nu} \to \frac{1}{2}\left(\frac{\phi_0}{2\pi|\Delta|\xi}\right)^2\left(j_{\mathrm{s},\mu}j_{\mathrm{s},\nu} - \frac{\delta_{\mu\nu}\boldsymbol{j}_{\mathrm{s}}^2}{2}\right), \tag{S33}$$

which coincides with the momentum flux tensor in the London equation [11]. We then finally identify $-\rho_{\mathrm{s}}\boldsymbol{Q}$ in the first term in the left-hand side with the superfluid component of the momentum density. We see that this term has the same sign as that of $\rho_{\mathrm{s}}\boldsymbol{j}_{\mathrm{s}}$. When $\lambda < 0$, the momentum density is antiparallel to the electric current density $\boldsymbol{j}_{\mathrm{s}}$ and thus the condensate corresponds to the positive electron density and the dynamics is similar to the electron motion. When $\lambda > 0$, on the other hand, the momentum density is parallel to the electric current density $\boldsymbol{j}_{\mathrm{s}}$ and thus the condensate corresponds to deficit of electron density and the dynamics is similar to the hole motion. In this section, we argue that the dynamics of the condensate described by the TDGL equation is similar to that of electron (hole) when λ is negative (positive) on the basis of analogy with Schrödinger equation, Eq. (S27b), and Eq. (S30).

Our expectation on the relation between the sign of the Hall conductivity and that of λ is consistent with the results of the Hartree approximation.

Supplementary Note 9: Comparison of theory and experiment

We examined whether the theoretical expressions of the conductivities [Eqs. (S21) and (S22)] and the Hall angle [Eq. (S26)] can explain the experimentally observed temperature and field dependence of ρ_{xx} and ρ_{yx} (Figs. 2a and 2c) and the concentration dependence of the Hall angle (Fig. 3b). The phenomenological parameters [T^*, $\partial T^*/\partial E_{\mathrm{F}}$, T_{c}, $B_{\mathrm{c2}}(0)$, σ_{xx}^{N}, $\sigma_{yx}^{\mathrm{N}}/B$] were obtained from the present and previous [1] experiments (Supplementary Table 2), and the dimensionless fluctuation interaction β was set by hand. We fitted the previous data of $T^*(E_{\mathrm{F}})$ [1] with a function $f(z) := c_1/(1+c_2 z^{c_3})$ (Supplementary Fig. 5), where the best-fitted parameters are $(c_1, c_2, c_3) = (97.8, 0.0888, 0.521)$, and we extrapolated the fitting curve to obtain T^* and $\partial T^*/\partial E_{\mathrm{F}}$ for the E_{F} values corresponding to $x = 0.0040$ and 0.47 in the present experiment. Note that T^* is estimated smaller than T_{c} for $x = 0.47$ simply due to the curve fitting to a few data points (Supplementary Fig. 5), which will not qualitatively affect the outcomes, though σ_{xx}^{V} and σ_{yx}^{V} may be overestimated. T_{c} and B_{c2} were obtained from the resistance measurement as the point for half of the normal resistance. σ_{xx}^{N} and $\sigma_{yx}^{\mathrm{N}}/B$ were determined from the experimental data at 30 K. In Fig. 4c, we plotted Θ_{H} for low enough temperatures, based on the fitting curve of $T^*(E_{\mathrm{F}})$ (Supplementary Fig. 5) and $\tan\Theta_{\mathrm{H}} \simeq -(4/\pi)\partial T^*/\partial E_{\mathrm{F}}$ [Eq. (S26)].

Using the parameters in Supplementary Table 2, we calculated ρ_{xx} and ρ_{yx} based on Eqs. (S21) and (S22), without cutoff ($c/h \to \infty$) for simplicity. The obtained temperature and field dependence of resistivity is shown in Supplementary Figs. 6 ($x = 0.004$) and 7 ($x = 0.47$) for $\beta = 10^{-4}$, 10^{-3}, and 10^{-2}. For $x = 0.004$ (Supplementary Fig. 6), the experimentally observed sign reversal and positive peak of ρ_{yx} (Fig. 2a) are qualitatively reproduced by the theory, in a broad range of the fluctuation strength parameter β. The peaks of ρ_{yx} are quantitatively higher than the experimentally observed ones especially at high fields, which might be explained by theoretical overestimation of the fluctuation contribution at high fields as known for the fluctuation-induced diamagnetism [12, 13] (see also [14, 15] for the overestimation of the fluctuation effects without introducing a cutoff in the GL formalism). On the other hand, for $x = 0.47$ (Supplementary Fig. 7), ρ_{yx} does not show a clear peak regardless of β, consistently with the experimental results (Fig. 2c). More quantitative comparison will require calculations starting from a microscopic Hamiltonian (e.g., [16]). For Figs. 4a and 4b, we chose $\beta = 10^{-3}$ (Supplementary Fig. 6, center) and $\beta = 10^{-4}$ (Supplementary Fig. 7, left), respectively.

Supplementary Table 2. **Parameters used to produce Figs. 4a–c in the main text.**

x	E_{F} (K)	T^*(K)	$\partial T^*/\partial E_{\mathrm{F}}$	T_{c}(K)
0.0040	102	49.1	−0.125	16.8
0.47	1.28×10^4	7.35	-2.76×10^{-4}	11.4

x	$B_{\mathrm{c2}}(0)$(T)	$\sigma_{xx}^{\mathrm{N}}(\Omega^{-1}\mathrm{cm}^{-1})$	$\sigma_{yx}^{\mathrm{N}}/B$ $(\Omega^{-1}\mathrm{cm}^{-1}T^{-1})$
0.0040	5.95	670	4.52
0.47	0.79	6.57×10^4	547

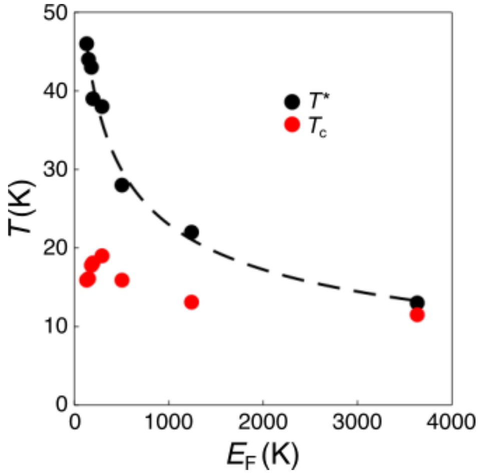

Supplementary Figure 5. **Gap-opening temperature T^* and superconducting critical temperature T_c as previously established[1].**
The black dashed line is the fitting curve of $T^*(E_F)$.

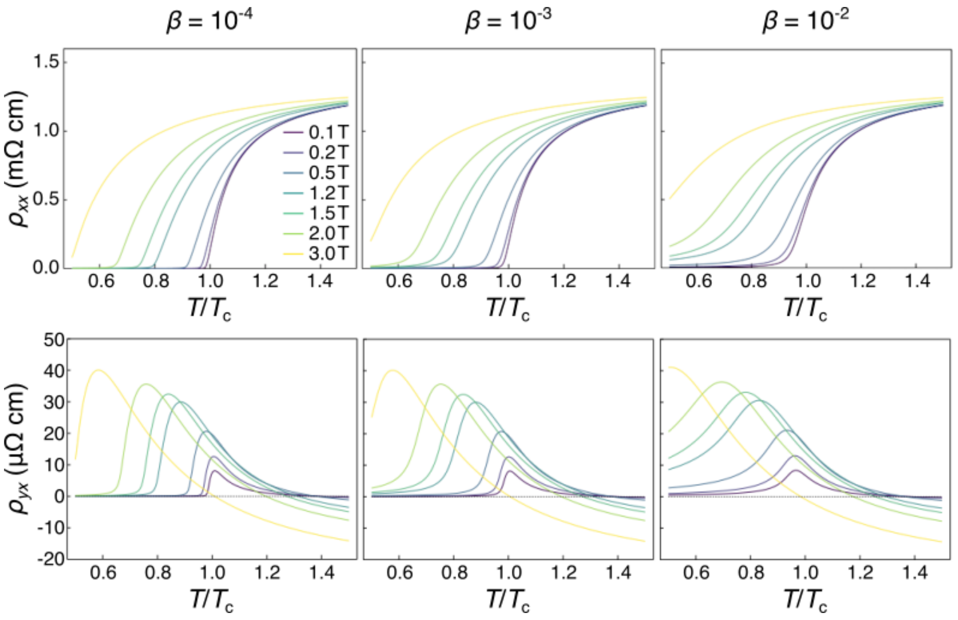

Supplementary Figure 6. **Theoretical longitudinal and transverse resistivities for $x = 0.0040$.**
The left, center, and right panels correspond to $\beta = 10^{-4}$, 10^{-3}, and 10^{-2}, respectively. Other parameters are summarized in Table S2.

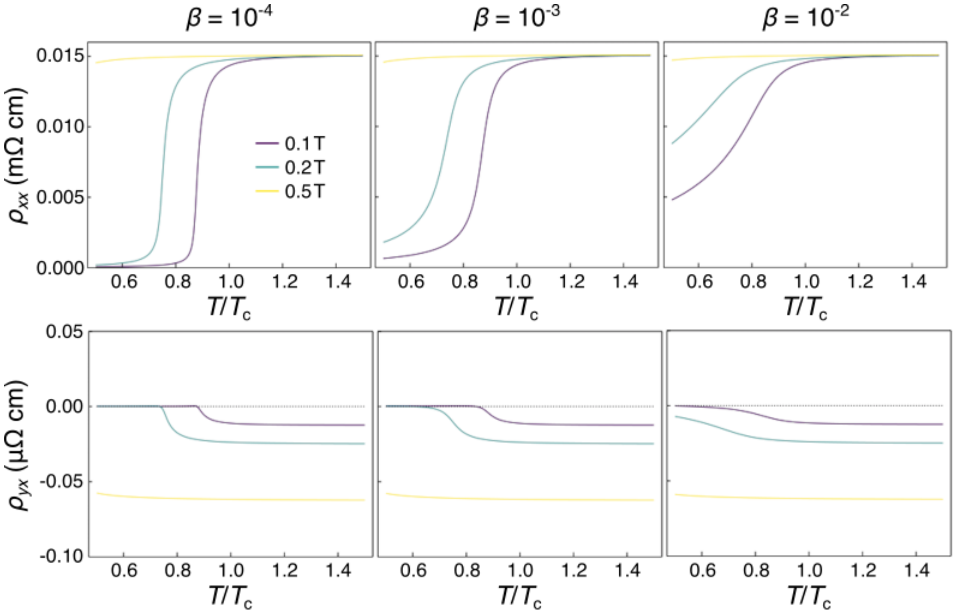

Supplementary Figure 7. **Theoretical longitudinal and transverse resistivities for $x = 0.47$.** The left, center, and right panels correspond to $\beta = 10^{-4}$, 10^{-3}, and 10^{-2}, respectively. Other parameters are summarized in Table S2.

Supplementary Note 10: Mean-field critical temperature T^*

We discuss the difference between $\partial T^*/\partial\mu$ and $\partial T^*/\partial E_{\rm F}$ for the 2D Fermi gas model (S39) within the mean-field approximation. Using the two-particle binding energy $E_{\rm B}$ instead of the coupling constant g, we obtain the equation to determine T^* for a given μ [17, 18]:

$$\int_0^\infty {\rm d}\varepsilon \left[\frac{1}{2\varepsilon + E_{\rm B}} - \frac{1}{2(\varepsilon-\mu)}\tanh\left(\frac{\varepsilon-\mu}{2T^*}\right)\right] = 0. \tag{S34}$$

Note that $\Delta_0 = \sqrt{2E_{\rm B}E_{\rm F}}$ within the mean-field approximation [18], where Δ_0 is the superconducting gap amplitude at zero temperature. For $\mu > 0$, we can rewrite Eq. (S34) as [19]

$$C_0 + \ln\frac{E_{\rm B}}{4T^*} + \tanh\left(\frac{\mu}{2T^*}\right)\ln\left(\frac{\mu}{2T^*}\right) - \int_0^{\mu/2T^*} {\rm d}x \frac{\ln x}{(\cosh x)^2} = 0, \tag{S35}$$

where $C_0 := -\int_0^\infty {\rm d}x \ln x/(\cosh x)^2 = 0.81878...$ Considering a small change in μ and the resulting change in T^* in Eq. (S35), we can obtain

$$\frac{\partial T^*}{\partial\mu} = \frac{\tanh(\mu/2T^*)}{1+\tanh(\mu/2T^*)}\frac{T^*}{\mu}. \tag{S36}$$

Then, we regard T^* as a function of $E_{\rm F}$ $(= \pi\rho/m)$, where ρ is the particle density, and use the formula of μ for the 2D Fermi gas [17–19]:

$$\mu = T^*\ln({\rm e}^{E_{\rm F}/T^*} - 1) =: \mu(T^*, E_{\rm F}). \tag{S37}$$

Considering a small change in $E_{\rm F}$ and the resulting change in T^* in Eqs. (S35) and (S37), we can obtain

$$\frac{\partial T^*}{\partial E_{\rm F}} = \left[\frac{E_{\rm F}}{T^*} + \frac{{\rm e}^{(\mu-E_{\rm F})/T^*}}{\tanh(\mu/2T^*)}\frac{\mu}{T^*}\bigg|_{\mu=\mu(T^*,E_{\rm F})}\right]^{-1}. \tag{S38}$$

To summarize, we obtain T^* and $\mu(T^*, E_{\rm F})$ by solving Eqs. (S35) and (S37) simultaneously, and then we can determine $\partial T^*/\partial\mu|_{\mu=\mu(T^*,E_{\rm F})}$ and $\partial T^*/\partial E_{\rm F}$ from Eqs. (S36) and (S38), respectively. In Supplementary Figs. 8(a-c), we show the calculated T^*, $\mu(T^*, E_{\rm F})$, $\partial T^*/\partial\mu|_{\mu=\mu(T^*,E_{\rm F})}$, and $\partial T^*/\partial E_{\rm F}$ as a function of $\Delta_0/E_{\rm F}$. We find that the ratio of $\partial T^*/\partial\mu|_{\mu=\mu(T^*,E_{\rm F})}$ to $\partial T^*/\partial E_{\rm F}$ is close to 1 even for moderate values of $\Delta_0/E_{\rm F}$ [Supplementary Fig. 8(d)] such

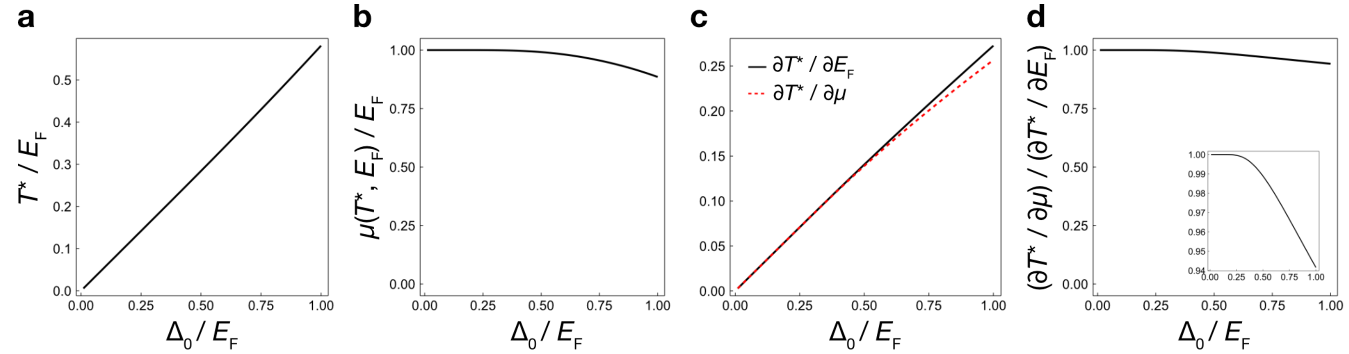

Supplementary Figure 8. **Several quantities at the mean-field critical temperature.** (**a**) The mean-field critical temperature T^* and (**b**) the chemical potential at T^* as a function of the zero-temperature gap Δ_0 divided by the Fermi energy E_F. (**c**) $\partial T^*/\partial E_\mathrm{F}$ and $\partial T^*/\partial\mu|_{\mu=\mu(T^*,E_\mathrm{F})}$, as well as (**d**) the ratio of them, as a function of Δ_0/E_F. For (**d**), an enlarged plot is shown in the inset.

as $\Delta_0/E_\mathrm{F} = 0.4$, which is a typical value observed in $\mathrm{Li}_x\mathrm{ZrNCl}$ [1] (Supplementary Fig. 1).

Supplementary Note 11: Dilute Fermi gas model

If we further reduce the doping x of $\mathrm{Li}_x\mathrm{ZrNCl}$ than in the present experiment, the distance between conduction electrons can become larger than the range of the effective attractive interaction. Then, the system may be described by the 2D Fermi gas model with a contact attractive interaction ($g > 0$):

$$H := \sum_{\sigma=\uparrow,\downarrow} \int \mathrm{d}^2\boldsymbol{r}\, \psi_\sigma^\dagger(\boldsymbol{r}) \left(-\frac{\boldsymbol{\nabla}^2}{2m}\right) \psi_\sigma(\boldsymbol{r}) - g \int \mathrm{d}^2\boldsymbol{r}\, \psi_\uparrow^\dagger(\boldsymbol{r})\psi_\downarrow^\dagger(\boldsymbol{r})\psi_\downarrow(\boldsymbol{r})\psi_\uparrow(\boldsymbol{r}), \tag{S39}$$

where $\psi_\sigma(\boldsymbol{r})$ and $\psi_\sigma^\dagger(\boldsymbol{r})$ are the Fermion field operators. In this model, the crossover from the BCS regime to the BEC regime occurs as the Fermion density is decreased [17, 19, 20]. Based on the expansion of the fluctuation propagator [21–24], we can derive the TDGL model corresponding to Eq. (S39) in the form of Eq. (S8). The coefficients of the TDGL model are now connected to the microscopic quantities as

$$\gamma = \frac{\pi}{4T^* c}\theta(\mu^*), \tag{S40}$$

$$\lambda = -\frac{1}{4T^* c}\mathrm{P}\int_{\mu^*/2T^*}^{\infty} \mathrm{d}x \frac{\tanh x}{x^2}, \tag{S41}$$

$$b = \frac{m}{8\pi c}\int_0^\infty \mathrm{d}\varepsilon \left[\frac{X}{(\varepsilon-\mu^*)^3} - \frac{Y}{2T^*(\varepsilon-\mu^*)^2}\right], \tag{S42}$$

$$\xi^2 = -\frac{1}{32\pi c}\int_0^\infty \mathrm{d}\varepsilon \left[\frac{X}{(\varepsilon-\mu^*)^2} - \frac{Y}{2T^*(\varepsilon-\mu^*)} + \frac{\varepsilon XY}{2(T^*)^2(\varepsilon-\mu^*)}\right], \tag{S43}$$

where $\theta(z)$ is the Heaviside step function, $\mathrm{P}(\cdots)$ means the Cauchy principal value, μ^* is the mean-field chemical potential at T^*, $X := \tanh[(\varepsilon-\mu^*)/2T^*]$, $Y := 1 - X^2$, $c := 1 + (E_\mathrm{F}/\mu^*)\exp[(E_\mathrm{F}-\mu^*)/T^*]\tanh(\mu^*/2T^*)$, and E_F is the Fermi energy.

In the BCS side (high density and $\mu^* > 0$), Eqs. (S40) and (S41) lead to $\gamma > 0$ and $\lambda < 0$, and thus $\Theta_\mathrm{H} < 0$ from Eq. (S25). Especially, deep in the BCS regime, where $T^* \ll \mu^* \simeq E_\mathrm{F}$, we can see $c \simeq 2$, $\gamma \simeq \pi/8T^*$, $\lambda \simeq -1/4E_\mathrm{F}$, and thus $\Theta_\mathrm{H} \simeq -2T^*/\pi E_\mathrm{F}$. Note that the sign of Θ_H (negative) is opposite to the experimentally observed values (positive), which suggests that the contact interaction in Eq. (S39) cannot explain the present doping range, and finite-range interactions may be important as indicated by a recent work [16].

In the BEC side (low density and $\mu^* < 0$), we see $\gamma = 0$ from Eq. (S40), and thus $\sigma_{xx}^\mathrm{V} = 0$ according to Eq. (S21). For low temperatures satisfying $\epsilon + h \ll h$, the normal-state conductivity, σ_{ab}^N, is expected to be negligible compared to $\sigma_{yx}^\mathrm{V} \simeq -2\pi\lambda T/[{\phi_0}^2(\epsilon+h)]$ [Eq. (S24)]. Thus, the Hall angle will be $\Theta_\mathrm{H} = -\arctan[(\sigma_{yx}^\mathrm{N} + \sigma_{yx}^\mathrm{V})/\sigma_{xx}^\mathrm{N}] \simeq -\pi/2$

for sufficiently low temperatures. Note that the conductivity calculated from the TDGL expansion combined with the Hartree approximation [Eqs. (S21) and (S22)] can become worse toward the BEC side since the separation between T^* and T_c is expected to be remarkable in the BEC side. Nevertheless, we expect that $\Theta_H \sim -\pi/2$ at low temperatures ($T \ll |\mu|$) where the dissipation is negligible. According to the Bogoliubov-de Gennes equations in the low-temperature limit, where only a few quantized levels exist in each vortex core [25] and thus those states are hardly scattered by impurities and phonons. Further, spatially extended quasiparticles and collective modes have gapped spectra and thus the scattering between the vortex and thermally excited quasiparticles or collective modes are negligible. Consequently, the momentum transfer between the vortex and the background such as impurities, phonons and quasiparticles are negligible and vortex motion is similar to that in an ideal fluid, where a vortex flows with the velocity same as that of the fluid. In charged superconductors, the macroscopic vortex flow with averaged velocity $\boldsymbol{v}_v$ induces spatially averaged electric field $\boldsymbol{E} = \boldsymbol{B} \times \boldsymbol{v}_v$ with the spatially averaged magnetic field $\boldsymbol{B}$ [26]. When vortices flow parallel to the superflow, the transport current $\boldsymbol{J}_{tr}$ and $\boldsymbol{v}_v$ are anti-parallel and thus $\boldsymbol{E}$ and $\boldsymbol{J}_{tr} \times \boldsymbol{B}$ are parallel, i.e. $\sigma_{xx} = 0$ and $\sigma_{xy} < 0$ for $\boldsymbol{B}$ parallel to z-axis. It then follows that $\Theta_H = -\pi/2$.

---

[1] Y. Nakagawa, Y. Kasahara, T. Nomoto, R. Arita, T. Nojima, and Y. Iwasa, Science **372**, 190 (2021).
[2] L. Sobirey, H. Biss, N. Luick, M. Bohlen, H. Moritz, and T. Lompe, https://arxiv.org/abs/2106.11893 (2021).
[3] M. G. Ries, A. N. Wenz, G. Zürn, L. Bayha, I. Boettcher, D. Kedar, P. A. Murthy, M. Neidig, T. Lompe, and S. Jochim, Phys. Rev. Lett. **114**, 230401 (2015).
[4] Y. Nakagawa, Y. Saito, T. Nojima, K. Inumaru, S. Yamanaka, Y. Kasahara, and Y. Iwasa, Phys. Rev. B **98**, 064512 (2018).
[5] Y. Kasahara, T. Kishiume, T. Takano, K. Kobayashi, E. Matsuoka, H. Onodera, K. Kuroki, Y. Taguchi, and Y. Iwasa, Phys. Rev. Lett. **103**, 077004 (2009).
[6] S. J. Hagen, A. W. Smith, M. Rajeswari, J. L. Peng, Z. Y. Li, R. L. Greene, S. N. Mao, X. X. Xi, S. Bhattacharya, Q. Li, and C. J. Lobb, Phys. Rev. B **47**, 1064 (1993).
[7] S. Ullah and A. T. Dorsey, Phys. Rev. B **44**, 262 (1991).
[8] E. Abrahams, R. Prange, and M. Stephen, Physica **55**, 230 (1971).
[9] Y. Kato and C.-K. Chung, J. Phys. Soc. Jpn. **85**, 033703 (2016).
[10] M. Tinkham, Phys. Rev. Lett. **13**, 804 (1964).
[11] F. London, *Superfluids I—Macroscopic Theory of Superconductivity* (Wiley, New York, 1950).
[12] P. A. Lee and M. G. Payne, Phys. Rev. Lett. **26**, 1537 (1971).
[13] P. A. Lee and M. G. Payne, Phys. Rev. B **5**, 923 (1972).
[14] C. Carballeira, J. Mosqueira, A. Revcolevschi, and F. Vidal, Phys. Rev. Lett. **84**, 3157 (2000).
[15] F. Vidal, C. Carballeira, S. R. Currás, J. Mosqueira, M. V. Ramallo, J. A. Veira, and J. Viña, EPL **59**, 754 (2002).
[16] T. Shi, W. Zhang, and C. A. R. Sá de Melo, https://arxiv.org/abs/2106.10010 (2021).
[17] S. S. Botelho and C. A. R. Sá de Melo, Phys. Rev. Lett. **96**, 040404 (2006).
[18] L. Salasnich, P. A. Marchetti, and F. Toigo, Phys. Rev. A **88**, 053612 (2013).
[19] V. P. Gusynin, V. M. Loktev, and S. G. Sharapov, J. Exp. Theor. Phys. **88**, 685 (1999).
[20] D. M. Eagles, Phys. Rev. **186**, 456 (1969).
[21] C. A. R. Sá de Melo, M. Randeria, and J. R. Engelbrecht, Phys. Rev. Lett. **71**, 3202 (1993).
[22] Y. Yanase and K. Yamada, J. Phys. Soc. Jpn. **68**, 2999 (1999).
[23] J. Stajic, A. Iyengar, Q. Chen, and K. Levin, Phys. Rev. B **68**, 174517 (2003).
[24] L. Han and C. A. R. Sá de Melo, New J. Phys. **13**, 055012 (2011).
[25] R. Sensarma, M. Randeria, and T.-L. Ho, Phys. Rev. Lett. **96**, 090403 (2006).
[26] B. Josephson, Phys. Lett. **16**, 242 (1965).
